# Supplementary material for: CRISPR-Cas9 screening reveals TM9SF2 knockout as a solution to HEK293 cell aggregation for improved AAV production
Source: Mol Ther Adv. 2026 Jun 11;34(3):201777. doi: 10.1016/j.omta.2026.201777 (PMC13316624; doi:10.1016/j.omta.2026.201777)
Supplement: Document S1. Figures S1–S6 and Tables S1–S4 [file mmc1.pdf]

OMTA, Volume *34*

Supplemental information

CRISPR-Cas9 screening reveals *TM9SF2* knockout as a solution to HEK293 cell aggregation for improved AAV production

Sungje Park, Seunghyeon Shin, Gyucheol Han, Yubin Won, Sang Yoon Lee, David Razafsky, Henry George, and Gyun Min Lee

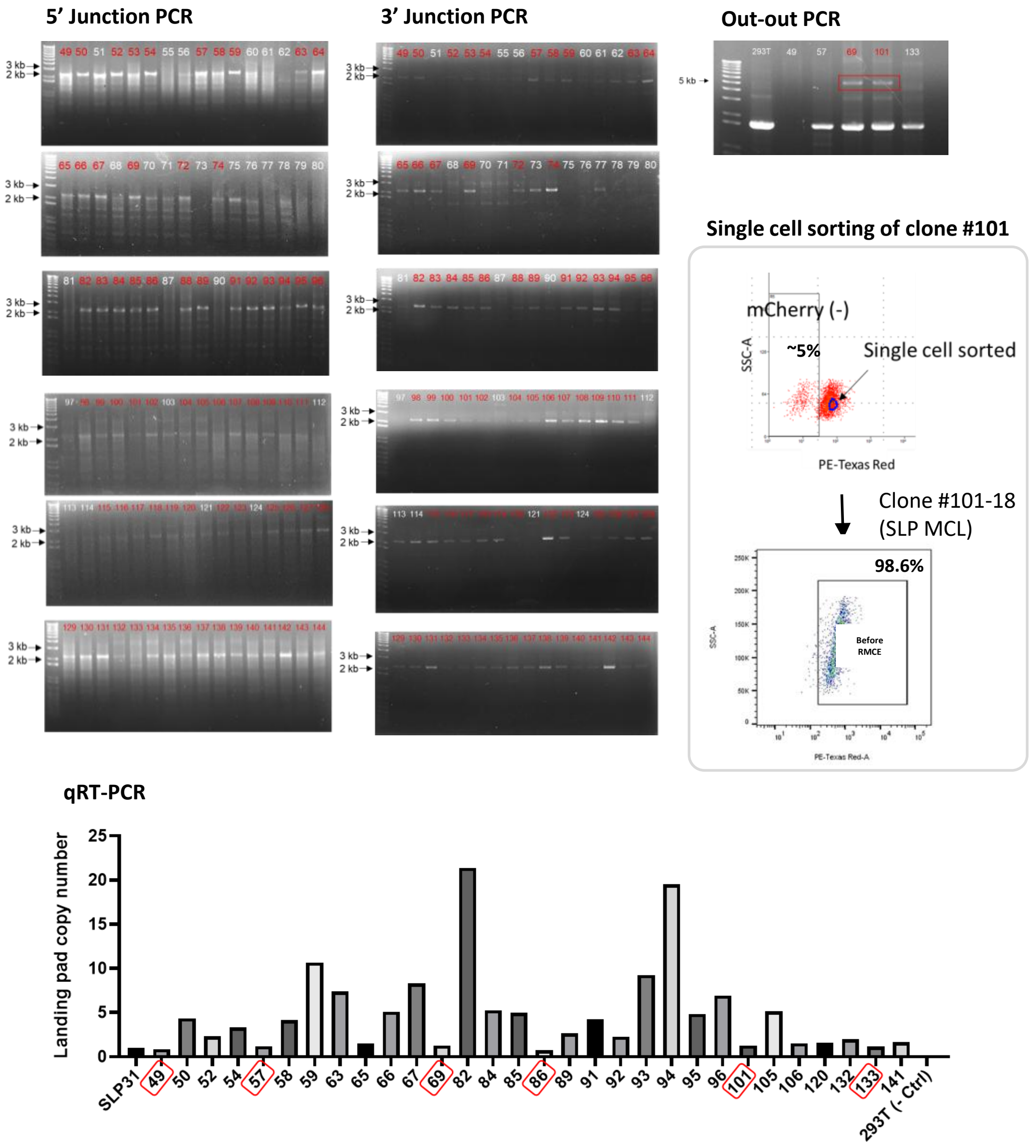

Figure S1. Targeted integration at the *ROSA26* locus was verified in 96 clones through 5'/3'-junction polymerase chain reaction (PCR), with positive clones highlighted in red. Following junction PCR, quantitative real-time PCR (qRT-PCR) was performed to confirm single-copy integration of the landing pad, using the reference single landing pad (SLP)31 clone for comparison. Among SLP clones identified, two clones were positive for out-out PCR. Clone #101 was initially selected based on its superior growth profile and mCherry enrichment; however, approximately 5% of cells were mCherry-negative. To improve population homogeneity, the mCherry-positive fraction was single-cell sorted, and subclones were evaluated. Clone #101-18 (SLP101-18) was selected as the master cell line (MCL) for further applications, as it exhibited a reduced mCherry-negative fraction (~1–2%) while maintaining robust growth.

#101-18 (D11)
NTC

#101-18 (D11)
RMCE

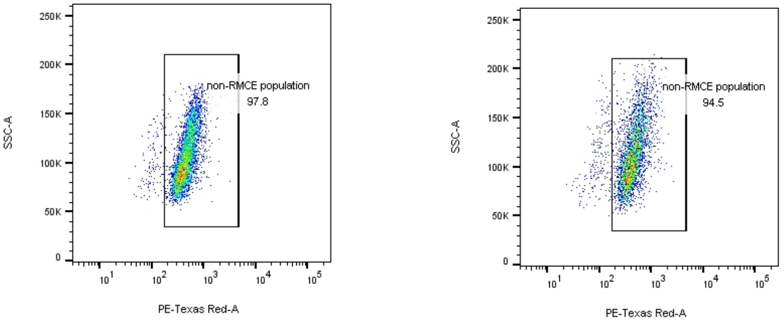

Non-RMCE population: 97.8 %

Non-RMCE population: 94.5 %

RMCE efficiency: 3.3 %

Figure S2. RMCE efficiency test of single landing pad master cell line (clone #101-18). Cells were transfected with gRNA library plasmid and NP-C-NLS-Bxb1 plasmid at a 3:1 weight ratio using PEIMAX as previously described (1). 48 hours-post transfection (HPT), cells were passaged every 3 days with fresh media. RMCE efficiency was measured at day 11 after transfection.

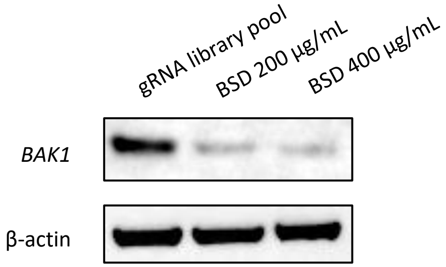

Figure S3. Knockout efficiency test of HEK293T cell-based gRNA library. For transfection, cells were seeded at a concentration of 1 × 10^6 cells/mL in fresh medium and transfected with gRNA(*BAK1*)-Cas9-T2A-BSD vector and PEIMAX (Polysciences, Warrington, PA) as previously described (1). 48 HPT, cells were selected with 200 and 400 µg/mL blasticidin for 3 days. After selection, cells were recovered for 4 passages. β-actin was used as a loading control.

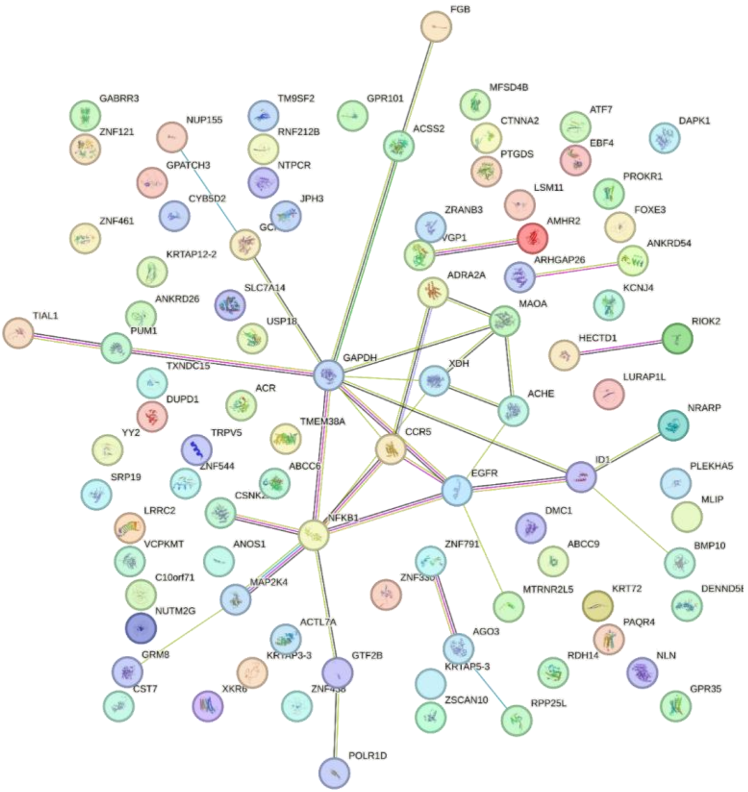

Figure S4. Schematic illustration of protein-protein interaction by STRING analysis of 93 genes (≥2 sgRNA per gene).

HEK293T

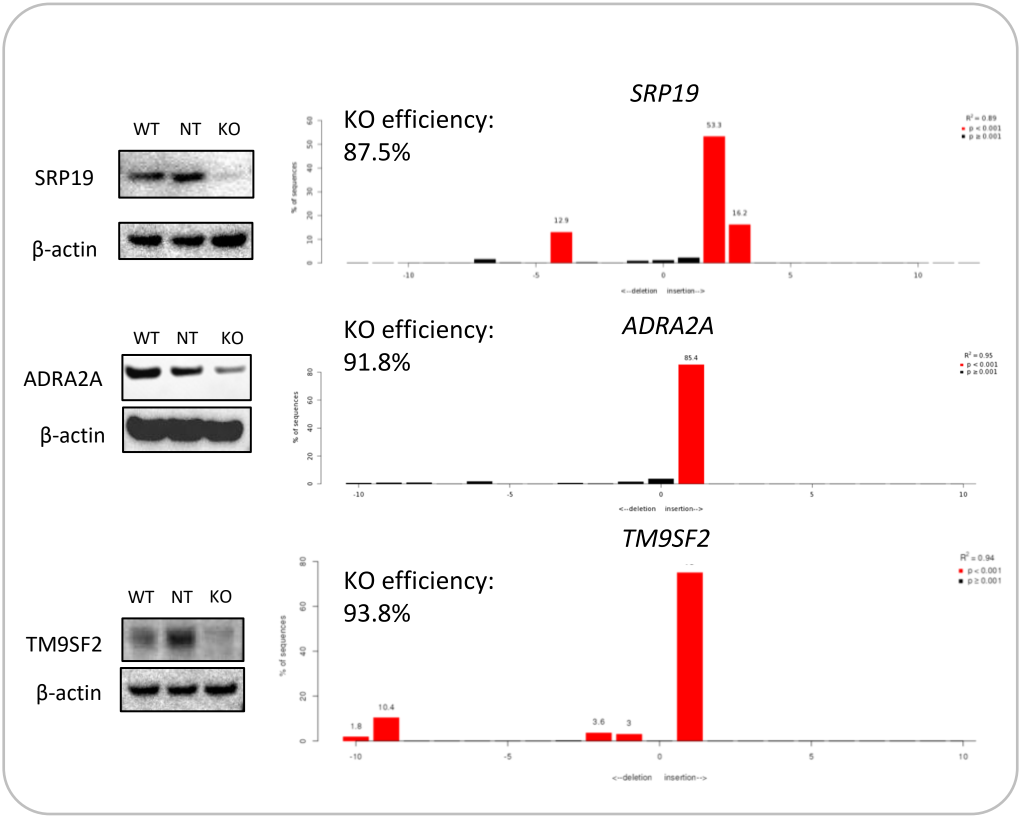

HEK293

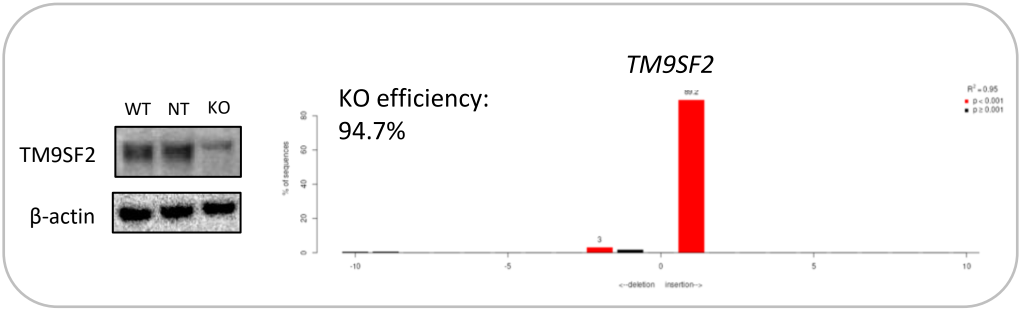

Figure S5. Evaluation of genome editing efficiency in knockout cell pools through western blotting and tracking of indels by decomposition (TIDE). Western blotting and TIDE analysis were conducted as previously described.(40)

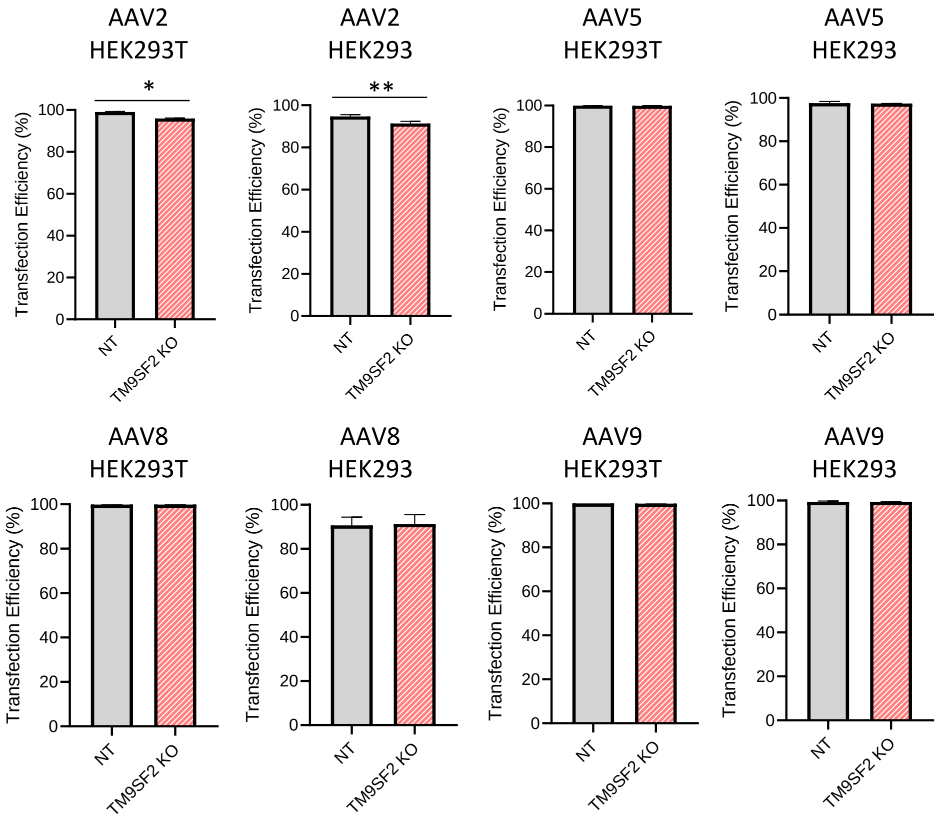

Figure S6. Transfection Efficiency During AAV Production

Table S1. Top 10 candidate genes from 93 significant genes (≥2 sgRNAs per gene).

Gene	Gene name	aRRA	# sgRNAs	# signif. sgRNAs
SRP19	signal recognition particle 19	3.78E-05	4	2
ADRA2A	adrenoceptor alpha 2A	7.55E-05	4	2
EGFR	epidermal growth factor receptor	7.58E-05	4	3
TM9SF2	transmembrane 9 superfamily member 2	0.0001	4	2
ZNF791	zinc finger protein 791	0.000121	4	2
ACHE	acetylcholinesterase	0.000138	4	2
PROKR1	prokineticin receptor 1	0.000163	4	2
TXNDC15	thioredoxin domain containing 15	0.000201	4	2
ACR	acrosin	0.000202	4	2
KRTAP5-3	keratin associated protein 5-3	0.00022	4	2

Table S2. Plasmid information.

Plasmid name	Description	Reference
ROSA26 LP donor	Landing pad donor plasmid targeting *ROSA26* locus	Shin et al., 2020
pU6-(BbsI)_CBh-Cas9-T2A-BFP vector	BFP-all-in-one CRISPR/Cas9 plasmid	Addgene plasmid #64323
pU6-(*ROSA26*)_CBh-Cas9-T2A-BFP	BFP-all-in-one CRISPR/Cas9 plasmid targeting *ROSA26* site	Cloned in this study
PuroR-attB-Esp3I	gRNA library backbone plasmid,<br>Plasmid for RMCE efficiency test	Kim et al., 2023
NP-C-NLS-Bxb1	Bxb1 recombinase vector with nucleoplasmin signal sequence in C terminus	Shin et al., 2021
gRNA(*BAK1*)-Cas9-T2A-Blast	KO efficiency test for deteriminig BSD concentration	Cloned in our previous study (Manuscript in revision)
Cas9-Blast	Cas9 expression plasmid that includes a blasticidin resistance gene.	Xiong et al., 2021<br>Kim et al., 2023
pSpCas9(BB)-T2A-Hygro	Hygro-all-in-one CRISPR/Cas9 plasmid	Addgene plasmid # 127763
gRNA(*target gene*)-Cas9-T2A-Hygro	All-in-one CRISPR/Cas9 plasmid targeting candidate KO target gene	Cloned in this study
pHelper	A vector containing the helper genes (E2A, E4orf6, and VA) for AAV production	A kind gift from Stratagene
pAAV2/2	A vector containing the AAV2 Rep gene and AAV2 Cap gene	Addgene plasmid #104963
pAAV2/5	A vector containing the AAV2 Rep gene and AAV5 Cap gene	Addgene plasmid #104964
pAAV2/8	A vector containing the AAV2 Rep gene and AAV8 Cap gene	Addgene plasmid #112864
pAAV2/9n	A vector containing the AAV2 Rep gene and AAV9 Cap gene	Addgene plasmid #112865

LP, landing pad; RMCE, recombinase-mediated cassette exchange; NP, Nucleoplasmin; NLS, nuclear localization signal; Blast, blasticidin resistance gene; Hygro, hygromycin resistance gene

Table S3. Primer information.

**Junction PCR primers**	
5‘ junction fwd	AAACCGGACGGAGCCAT
5‘ junction rev	CCATGTTATCCTCCTCGCCC
3‘ junction fwd	ATTCCCAATACGAGGTCGCC
3‘ junction rev	AAGGCATGACCAAAGGGGAG
**Outout PCR primers**	
Outout fwd (Rosa26 LP Seq 2)	GCTGCAATCCTGAGGGA
Outout rev (3' junction rev)	AAGGCATGACCAAAGGGGAG
**qRT-PCR primers**	
qRT-PCR fwd (mCherry)	GACTACTTGAAGCTGTCCTTCC
qRT-PCR rev (mCherry)	CGCAGCTTCACCTTGTAGAT
ACTB fwd	CTGGAACGGTGAAGGTGACA
ACTB rev	AAGGGACTTCCTGTAACAACGCA
AAV qRT-PCR fwd	ATGGTGATGCGGTTTTGGCAG
AAV qRT-PCR rev	GGCGGAGTTGTTACGACATTTTGG
**NGS primers**	
NGS fwd (gDNA)	GCTTTATATATCTTGTGGAAAGGACGAAACACC
NGS rev (gDNA)	CCGACTCGGTGCCACTTTTTCAA

Table S4. sgRNA oligo information.

Oligo name	Sequence (5' – 3')
*ROSA26*Fwd	CACCGGTCGAGTCGCTTCTCGATTA
*ROSA26*Rev	AAACTAATCGAGAAGCGACTCGACC
*BAK1*Fwd	CACCGGTTGATGTCGTCCCCGATGA
*BAK1*Rev	AAACTCATCGGGGACGACATCAACC
SRP19Fwd	CACCGTCTTGCTTACCTTACTTATG
SRP19Rev	AAACCATAAGTAAGGTAAGCAAGAC
ADRA2AFwd	CACCGTGGTCGTTGATCTCGCAGCG
ADRA2ARev	AAACCGCTGCGAGATCAACGACCAC
EGFRFwd	CACCGGTCTGCGTACTTCCAGACCA
EGFRRev	AAACTGGTCTGGAAGTACGCAGACC
TM9SF2Fwd	CACCGACACTTACATAGAGTCACAA
TM9SF2Rev	AAACTTGTGACTCTATGTAAGTGTC
ZNF791Fwd	CACCGTCACGTGTACTCGAAAGGCT
ZNF791Rev	AAACAGCCTTTCGAGTACACGTGAC
ACHEFwd	CACCGTGTCCTCGTCTGGATCTATG
ACHERev	AAACCATAGATCCAGACGAGGACAC
PROKR1Fwd	CACCGAGTGCGCAGGTAGTTGACAG
PROKR1Rev	AAACCTGTCAACTACCTGCGCACTC
TXNDC15Fwd	CACCGGGACACCCAAGGCGATCACA
TXNDC15Rev	AAACTGTGATCGCCTTGGGTGTCCC
ACRFwd	CACCGAAGCGCCCACACGAAATGGG
ACRRev	AAACCCCATTTCGTGTGGGCGCTTC
KRTAP5-3Fwd	CACCGGCAGACAGGTACACAGCAGC
KRTAP5-3Rev	AAACGCTGCTGTGTACCTGTCTGCC

Table S5. Significant genes.

Table S6. GO stat.

Table S7. GO genes.

Table S8. KEGG stat.

Table S9. KEGG genes.
