# Supplementary material for: CRISPR-Cas9 screening reveals TM9SF2 knockout as a solution to HEK293 cell aggregation for improved AAV production
Source: Mol Ther Adv. 2026 Jun 11;34(3):201777. doi: 10.1016/j.omta.2026.201777 (PMC13316624; doi:10.1016/j.omta.2026.201777)
Supplement: Document S2. Article plus supplemental information [file mmc7.pdf]

Molecular Therapy
Advances
Original Article

CRISPR-Cas9 screening reveals *TM9SF2* knockout as a solution to HEK293 cell aggregation for improved AAV production

Sungje Park,[1] Seunghyeon Shin,[1] Gyucheol Han,[2] Yubin Won,[2] Sang Yoon Lee,[1] David Razafsky,[3] Henry George,[3] and Gyun Min Lee[1,2]

[1]Department of Biological Sciences, KAIST, Daejeon, Republic of Korea; [2]Graduate School of Engineering Biology, KAIST, Daejeon, Republic of Korea; [3]MilliporeSigma, St. Louis, MO, USA

The inherent tendency of human embryonic kidney (HEK) cells to aggregate in suspension culture poses a significant obstacle to the large-scale production of adeno-associated virus (AAV) vectors for gene therapy. To overcome this, we employed a virus-free CRISPR-Cas9 single gene knockout (KO) library in HEK293T cells to systematically identify key genetic regulators of cell aggregation. By serially passaging the KO library and selectively sub-culturing only the suspended (non-aggregated) cells, we enriched for genotypes that reduce aggregation. Next-generation sequencing identified *TM9SF2* as a key target, and its KO reduced aggregation by 53% and 28% in HEK293T and HEK293 cells, respectively. Transcriptomic analysis of *TM9SF2* KO cells showed enrichment in MAPK signaling, calcium signaling, and plasma membrane components, offering insights into the mechanisms of aggregation suppression. Importantly, *TM9SF2* KO did not compromise the genome titer, full capsid ratio, or infectivity of AAV5, AAV8, and AAV9, representative serotypes for secreted AAVs, in both HEK293T and HEK293 cells. These findings identify *TM9SF2* as a promising target to mitigate cell aggregation in HEK293-based systems, thereby facilitating scalable AAV vector production.

INTRODUCTION

Adeno-associated viruses (AAVs) have emerged as promising vectors for gene therapy due to their safety, versatile tropism, and ability to achieve efficient and sustained expression of transgenes.[1–4] Among the various AAV production platforms, the triple-transfection method remains the most widely used platform, offering flexibility, scalability, and ease of implementation.[1–4] This method involves the co-transfection of three plasmids (pHelper, pRepCap, and pAAV-GOI) into human embryonic kidney 293 (HEK293) cells, which stably express the adenoviral E1A and E1B genes essential for AAV replication and packaging.[1–4]

In small-scale adherent cultures, HEK293 cells are seeded in growth medium, followed by a medium change to transfection medium prior to plasmid transfection. After transfection, the medium is reverted to the growth medium. While this workflow is relatively straightforward in T-flasks with adherent HEK293 cells, it becomes impractical in large-scale suspension cultures, preferred for industrial production, due to the difficulty of performing medium changes. To streamline the process, a single medium is now commonly employed for both cell growth and transfection throughout the AAV production process.[1,4–7]

A major challenge in suspension-culture-based AAV manufacturing is the tendency of HEK293 cells to form aggregates. Despite initial dispersion, significant cell clumping often occurs over time, which compromises transfection efficiency. Although chemical disaggregation reagents could mitigate this issue, their use is limited as they interfere with plasmid uptake.[8–10] Moreover, cell aggregation adversely affects cell viability, growth kinetics, and overall AAV yield and quality. Therefore, engineering HEK293 cells to exhibit reduced aggregation in suspension culture is a compelling strategy to enhance production robustness.

However, identifying genetic determinants of cell aggregation is complex and labor-intensive, as aggregation is driven by multifactorial mechanisms. These include: (1) cell-cell adhesion molecules such as cadherins, integrins, immunoglobulin-like receptors, and selectins[11]; (2) interactions between the extracellular matrix (ECM) and cytoskeletal components[11]; (3) intracellular signaling pathways including PI3K-Akt, MAPK, and Wnt[11–20]; and (4) extracellular DNAs released from lysed cells.[21]

To address this challenge more systematically, we employed a genome-wide CRISPR-Cas9 knockout (KO) screen in HEK293 cells using a recombinase-mediated cassette exchange (RMCE) system. By serially passaging the single gene KO cell library and selectively sub-culturing only the suspended (non-aggregated) cells, we enriched for

Received 17 January 2026; accepted 9 June 2026;
https://doi.org/10.1016/j.omta.2026.201777.

Correspondence: Gyun Min Lee, Department of Biological Sciences, KAIST, Daejeon, Republic of Korea
E-mail: gyunminlee@kaist.ac.kr

© 2026 The Author(s).
Published by Elsevier Inc. on behalf of The American Society of Gene and Cell Therapy.
This is an open access article under the CC BY license (http://creativecommons.org/licenses/by/4.0/).

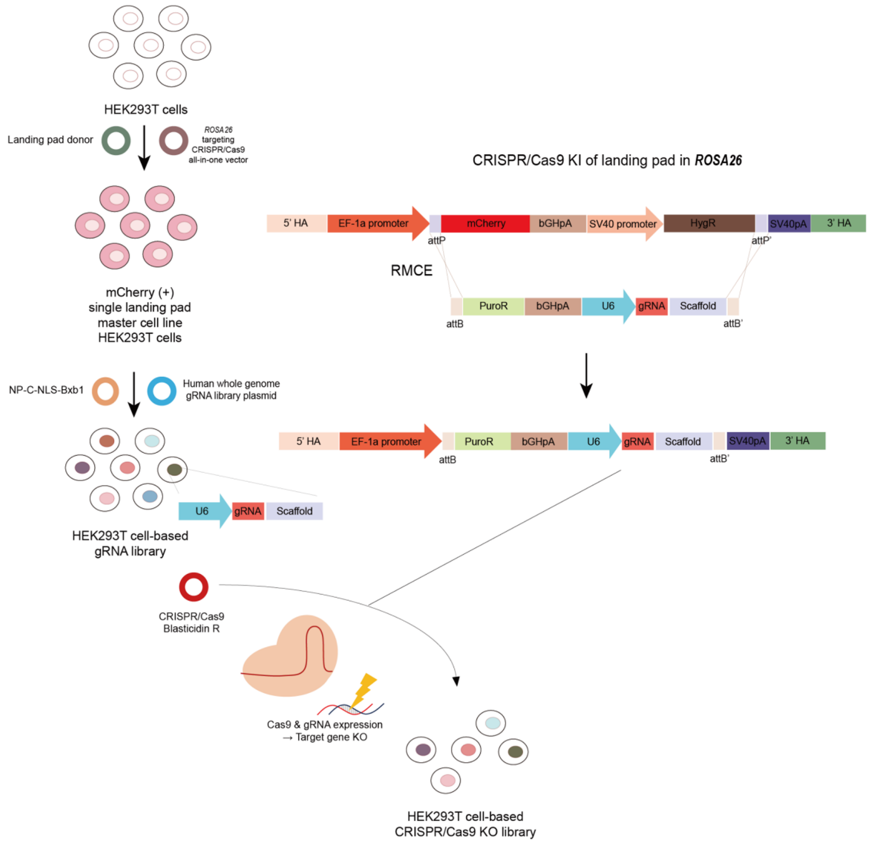

Figure 1. Schematic illustration of the development process for HEK293T cell-based CRISPR-Cas9 KO screening platform

genotypes associated with reduced aggregation. Enriched guide RNAs (gRNAs) were identified by next-generation sequencing (NGS), and candidate genes were validated in individual KO cell pools with respect to aggregation phenotype and AAV production performance in suspension culture.

RESULTS

Establishment of an RMCE-based HEK293T CRISPR-KO screening platform

A virus-free, RMCE-based CRISPR knockout screening platform was developed using HEK293T cells. As illustrated in Figure 1, a single landing pad (SLP) was integrated into the *ROSA26* safe harbor locus to generate a master cell line (MCL). This was achieved by co-transfecting HEK293T cells with a donor plasmid containing the landing pad and a CRISPR-Cas9 vector targeting *ROSA26*.

The landing pad construct included the mCherry reporter gene and two recombinase target sites (attP and attP*), enabling site-specific recombination. Of the 96 mCherry-positive clones isolated by flow cytometry, 70 clones were confirmed to have targeted landing pad integration via a 5′/3′ junction PCR analysis (Figure S1). The top 30 clones, selected based on growth characteristics, were further validated via real-time quantitative PCR (qPCR) and out-out PCR to confirm single-copy landing pad insertion. As detailed in Figure S1, clone #101-18, which demonstrated homogeneous mCherry expression and robust proliferation, was selected for subsequent CRISPR screening experiments and designated as the SLP MCL.

To introduce a gRNA library into SLP MCL using RMCE, the human Brunello CRISPR KO library comprising 77,441 gRNAs targeting 19,114 genes, including 1,000 non-targeting (NT) gRNAs, was first cloned into RMCE donor plasmids containing a gRNA expression cassette and recombinase target sites. SLP MCL was then co-transfected with the cloned gRNA library and NP-C-NLS-Bxb1 recombinase plasmids, ensuring a coverage of 500 cells per gRNA.

Starting on day 2 post-transfection, cells harboring the integrated gRNA library were enriched via puromycin selection for five passages (Figures 2A and 2B). The proportion of RMCE-positive cells increased from 3.3% to 98.4%, as indicated by the reduction in mCherry-positive cells (Figures 2C and S2). To verify the representation of the gRNA library, gRNA sequences were amplified from the genomic DNA and subjected to NGS. The sgRNA coverage and Gini coefficient of the cell-based gRNA library were 99.4% and 0.326, respectively, demonstrating sufficient representation with an even distribution (Figure 2D).

CRISPR-Cas9 KO library cells were generated through transient transfection with the Cas9-Blast plasmid, followed by effective enrichment using 400 μg/mL blasticidin selection for three days (Figure S3). After four passages without blasticidin, cells fully recovered and were subsequently used as CRISPR-Cas9 KO library cells for screening target genes associated with reduced cell aggregation (Figures 2E and 2F).

Screening of target genes associated with reduced cell aggregation

Non-aggregated cell populations in KO library cells were successfully isolated using conical tubes through differential sedimentation, as described in the materials and methods section. The screening strategy was designed to progressively enrich cells showing reduced aggregation, rather than to isolate physically identical cell populations. Aggregated cells rapidly sedimented within a few minutes, while non-aggregated cells remained suspended in the supernatant. To enrich for cells with reduced aggregation, only the non-aggregated cells were sub-cultured every three days, promoting progressive

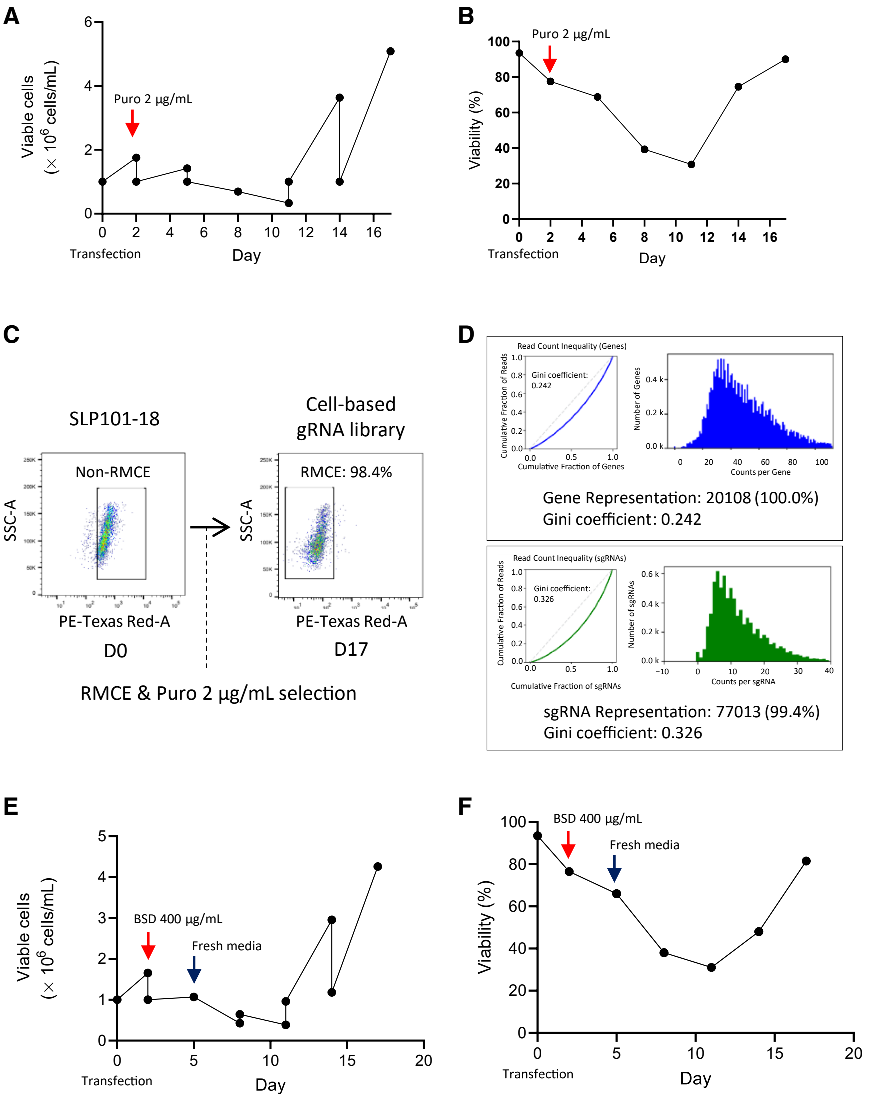

Figure 2. Development of HEK293T cell-based human whole genome CRISPR-Cas9 KO library

(A) Viable cell concentration (VCC) and (B) viability during the development of the RMCE cell pool. On day 0, MCL was transfected with NLS-Bxb1 recombinase and gRNA library plasmids to obtain the RMCE-positive cell pool selected with 2 μg/mL puromycin 48 h post-transfection (HPT). (C) RMCE-positive cell population after selection with 2 μg/mL puromycin. (D) Coverage and Gini-coefficient of gene and sgRNA of HEK293T cell-based gRNA library. (E) VCC and (F) viability during the development of the HEK293T cell-based human whole genome CRISPR-Cas9 KO library cell pool, selected with 400 μg/mL blasticidin, followed by fresh media replacement and recovery over 4 passages.

selection across successive passages. As a control, KO library cells were also passaged every three days, without selection (Figure 3A).

Over time, the cell growth profile of both control and experimental groups remained consistent (Figure 3B). However, distinct phenotypic differences in cell aggregation between the control and experimental groups became increasingly apparent (Figures 3C–3E). The proportion of non-aggregated cells (cells in suspension) relative to the total cell population increased steadily up to passage 3, after which it plateaued at passage 4. Furthermore, as shown in Figure 3D (control group) and Figure 3E (experimental group), the population of aggregated cells was substantially reduced following four rounds of selection in the experimental group. Subsequently, triplicate samples from both the control and experimental groups were subjected to NGS analysis to identify candidate genes associated with reduced cell aggregation.

To evaluate the distribution of gRNAs between the experimental and control groups, computational analysis was conducted using the platform-independent analysis of pooled screens using Python (PinAPL-Py).[22] The adjusted robust rank aggregation (αRRA) algorithm was employed to rank genes by integrating fold-change data across all gRNAs. To control the false discovery rate (FDR), p values were adjusted using the Benjamini-Hochberg correction.

Among 20,112 genes analyzed, including NT genes, 2,196 genes were identified as significant. Of these, 93 genes contained ≥2 sgRNAs per gene, indicating robust signal detection. The top 10 highest-scoring genes were selected as primary hits and are listed in Table S1.

Subsequently, Gene Ontology (GO) enrichment analysis was performed using the Database for Annotation, Visualization, and Integrated Discovery (DAVID), focusing on the 93 genes with ≥2 sgRNAs. The analysis revealed enrichment in pathways and molecular functions known to play critical roles in cell adhesion and aggregation, including calcium signaling, mechanical stimulus response, canonical Wnt signaling, MAPK cascade, receptor complex organization, and ion binding functions (Figure 4).[11–20] Additionally, an STRING-based protein-protein interaction (PPI) network analysis of the same set of 93 genes revealed significant interaction clusters, further supporting the functional connectivity among identified hits (Figure S4). This analysis was used to provide supportive evidence for functional relationships among the screened genes and was not used as a criterion for selecting targets for further validation.

Validation of candidate genes

To validate the screening results, KO suspension cell pools were generated for the top 10 highest-scoring candidate genes using

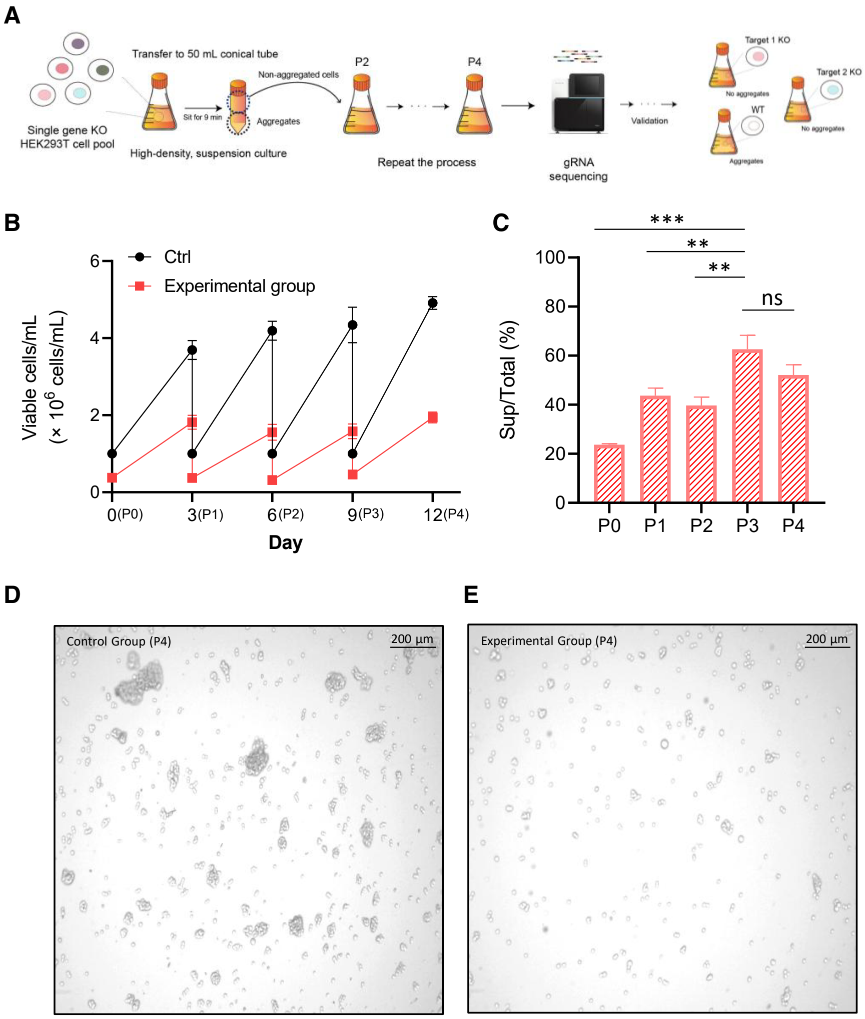

Figure 3. KO screening for finding KO gene targets to reduce cell aggregation

(A) Schematic illustration of the enrichment of non-aggregated cells in the CRISPR-Cas9 KO library cell pool. Non-aggregated cell population in the CRISPR-Cas9 KO library cell pool was separated and enriched from the KO library using their sedimentation differences, with aggregated cells settling faster in Falcon tubes. Cells were seeded at a concentration of 1.0×10^6 cells/mL in Erlenmeyer flasks with a 50 mL culture volume. After 2 days (P0), 48 mL of cell suspension was transferred to Falcon tubes, left to settle for 9 min, and 24 mL of the upper suspension was collected. Control cells were passaged every 3 days seeding at a concentration of 1×10^6 cells/mL in fresh medium. (B) Growth profiles of control and experimental groups over screening passages. (C) Proportion of non-aggregated cells (sup/total) across passages. (D) Microscope image (2.5×) of the control group after 4 passages of screening. (E) Microscope image (2.5×) of the experimental group after 4 passages of screening. Error bars represent the standard deviation calculated from three independent experiments. "ns" indicates nonsignificant value. $^{**}p < 0.01$, $^{***}p < 0.001$. Scale bars in all microscopic images are 200 μm.

all-in-one pSpCas9(BB)-T2A-Hygro plasmids targeting each gene. In parallel, a NT control HEK293T suspension cell pool was also established. All cell pools were cultured in suspension without transfection for three days in 6-well plates placed on a shaking incubator.

Cell aggregation, viable cell concentration (VCC), and viability were quantified using a CEDEX HiRES analyzer for assessment. Among the top 10 candidates, knockout of signal recognition particle 19 (*SRP19*), adrenoceptor alpha 2A (*ADRA2A*), and transmembrane 9 superfamily member 2 (*TM9SF2*) resulted in a significant reduction in cell aggregation (Figure 5A). The KO efficiencies for *SRP19*, *ADRA2A*, and *TM9SF2*, as determined by tracking of indels by decomposition (TIDE) analysis, were 87.5%, 91.8%, and 93.8%, respectively (Figure S5). Although minor differences were observed between TIDE analysis and western blot results, this likely reflects differences between DNA-level editing efficiency and protein-level expression. TIDE provides an estimate of total editing frequency, whereas western blot reflects the functional consequence of these edits at the protein level.

Compared with the NT control cell pool, cell aggregation was reduced by 23%, 24%, and 53% in the *SRP19*, *ADRA2A*, and *TM9SF2* KO cell pools, respectively. Importantly, VCC and viability of these KO pools were comparable to those of the control, indicating that disruption of these genes did not adversely affect cell growth (Figures 5B and 5C).

To further evaluate the most promising candidate, *TM9SF2*, additional KO and control suspension cell pools were generated in a different HEK293 cell line (HEK293 cells). The KO efficiency of *TM9SF2* in HEK293 cells was 94.7% (Figure S5). Consistent with results in HEK293T cells, *TM9SF2* KO in HEK293 cells led to a marked reduction in cell aggregation (Figure 5D), with a 28% decrease relative to the NT control.

The pronounced decrease in cell aggregation following *TM9SF2* KO in both HEK293T and HEK293 cells was also confirmed by microscopy (Figure 6). These findings demonstrate that the CRISPR-Cas9 KO screening successfully identified gene targets, particularly *TM9SF2*, that significantly reduce cell aggregation in HEK293-derived cell lines.

Transcriptomic analysis of the *TM9SF2* KO cell pool

Transcriptome re-sequencing identified 419 significant differentially expressed genes (DEGs) between the *TM9SF2* KO HEK293T cell pool and the NT HEK293T control, based on the criteria of $|fc| \geq 2$ and p value <0.05 (Figures 7A and 7B). These genes were subsequently analyzed using gProfiler for GO enrichment to identify statistically overrepresented biological functions. Enriched GO terms were

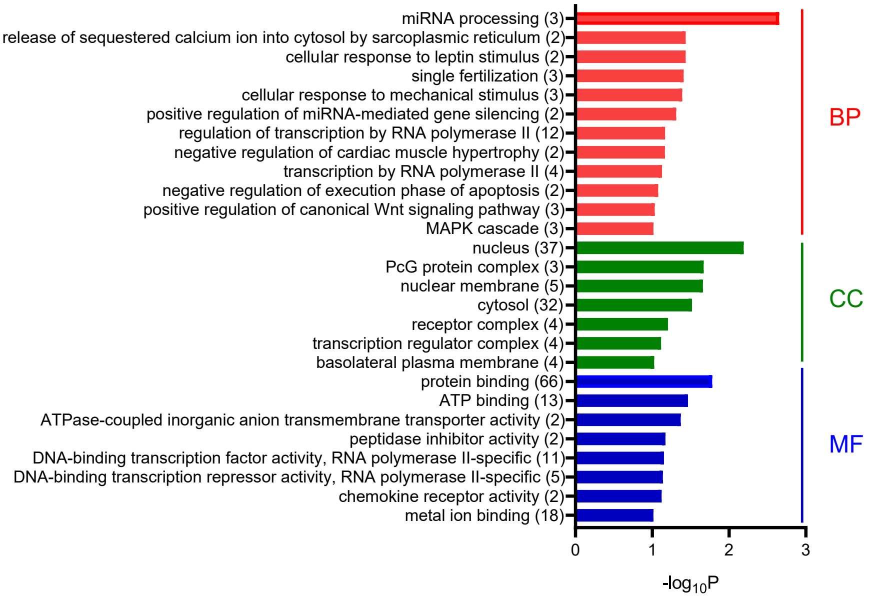

Figure 4. Functional annotation chart of enriched Gene Ontology terms across biological processes, cellular components, and molecular functions for 93 significant genes, with ≥2 sgRNAs per gene
Statistical analysis was conducted using default settings. BP, biological processes; CC, cellular components; MF, molecular functions.

determined based on adjusted *p* values and the proportion of DEGs assigned to each term (GeneRatio). Functional enrichment analysis of the DEGs was conducted across the biological process (BP), cellular component (CC), and molecular function (MF) categories. The top 10 enriched GO terms ranked by adjusted *p* value are shown separately for BP in Figure 7C and CC in Figure 7D. Because only three GO terms met the enrichment criteria in the MF category, the top 3 enriched GO terms are shown in Figure 7E. In the BP category, the top 10 enriched GO terms ranked by adjusted *p* value were primarily associated with cell clustering, tissue organization, and developmental processes (Figure 7C). In the CC category, the enriched terms were mainly related to membrane and ECM-associated components, including the extracellular region, plasma membrane, and ECM (Figure 7D). Within the MF category, enriched terms included calcium ion binding, monatomic ion channel activity, and monatomic ion transmembrane transporter activity (Figure 7E).

KEGG pathway enrichment analysis identified multiple signaling pathways associated with the DEGs in *TM9SF2* KO cells. The top 20 enriched pathways ranked by *p* value are shown in Figure 7F, among which PI3K-Akt signaling, MAPK signaling, calcium signaling, and cytoskeleton-related pathways were included. These pathways have previously been reported to participate in cellular processes such as cell adhesion, cytoskeletal dynamics, and intercellular communication.[11–20]

Assessment of *TM9SF2* knockout in AAV production

To evaluate the impact of *TM9SF2* KO on AAV production, genome titer, full capsid ratio, and infectivity were assessed across multiple AAV serotypes with distinct production kinetics. AAVs were produced in *TM9SF2* KO HEK293T and *TM9SF2* KO HEK293 cell pools using the triple transfection method as described in the materials and methods section. Genome titers and capsid titers were quantified by qPCR and ELISA, respectively, using crude AAV preparations. Although purified samples generally allow more accurate quantification, crude lysates are sufficient for relative comparison. Parallel cultures of NT control HEK293T and HEK293 suspension cell pools served as controls.

AAV2, representing the cell-associated AAV model, showed no differences in virus genome (VG) titer or full capsid ratio upon *TM9SF2* KO. However, *TM9SF2* KO led to a marked decrease in infectivity (Figures 8A and 8B). Infectivity was quantified as the ratio of transducing units (TU) to VG. The TU/VG ratios for *TM9SF2* KO HEK293T and HEK293 cells were $(2.2 \pm 0.9) \times 10^{-2}$ and $(5.8 \pm 0.5) \times 10^{-2}$, respectively, corresponding to just 20.5% and 51.8% of the respective NT controls. These results suggest that *TM9SF2* KO is not suitable for producing cell-associated AAV2, as it is associated with impaired functional infectivity despite preserved genome titers.

In contrast, *TM9SF2* KO did not impair the infectivity of secreted AAV serotypes (Figures 8C–8H). For AAV5, AAV8, and AAV9, selected as models for secreted AAVs, production outcomes varied by serotype, but *TM9SF2* KO had no detrimental effect on genome titer, full capsid ratio, or infectivity in either HEK293T or HEK293 cells.

Transfection efficiency during AAV production is summarized in Figure S6. For AAV2 production, although a statistically significant difference in transfection efficiency was observed, the magnitude of the difference was very small and was therefore not considered meaningful in this context. No significant differences in transfection efficiency were observed for the other serotypes.

DISCUSSION

The tendency of HEK293 cells to aggregate in suspension culture presents a major challenge in large-scale AAV manufacturing.[23,24] Despite its industrial relevance, limited research has been directed toward mitigating cell aggregation in HEK293 cells.[25] While it may seem rational to target genes involved in cell adhesion, this approach carries the risk of compromising cell viability and productivity as these genes play an important role in maintaining cellular integrity. To overcome this challenge, we employed a genome-wide CRISPR-Cas9 KO screen to identify gene targets

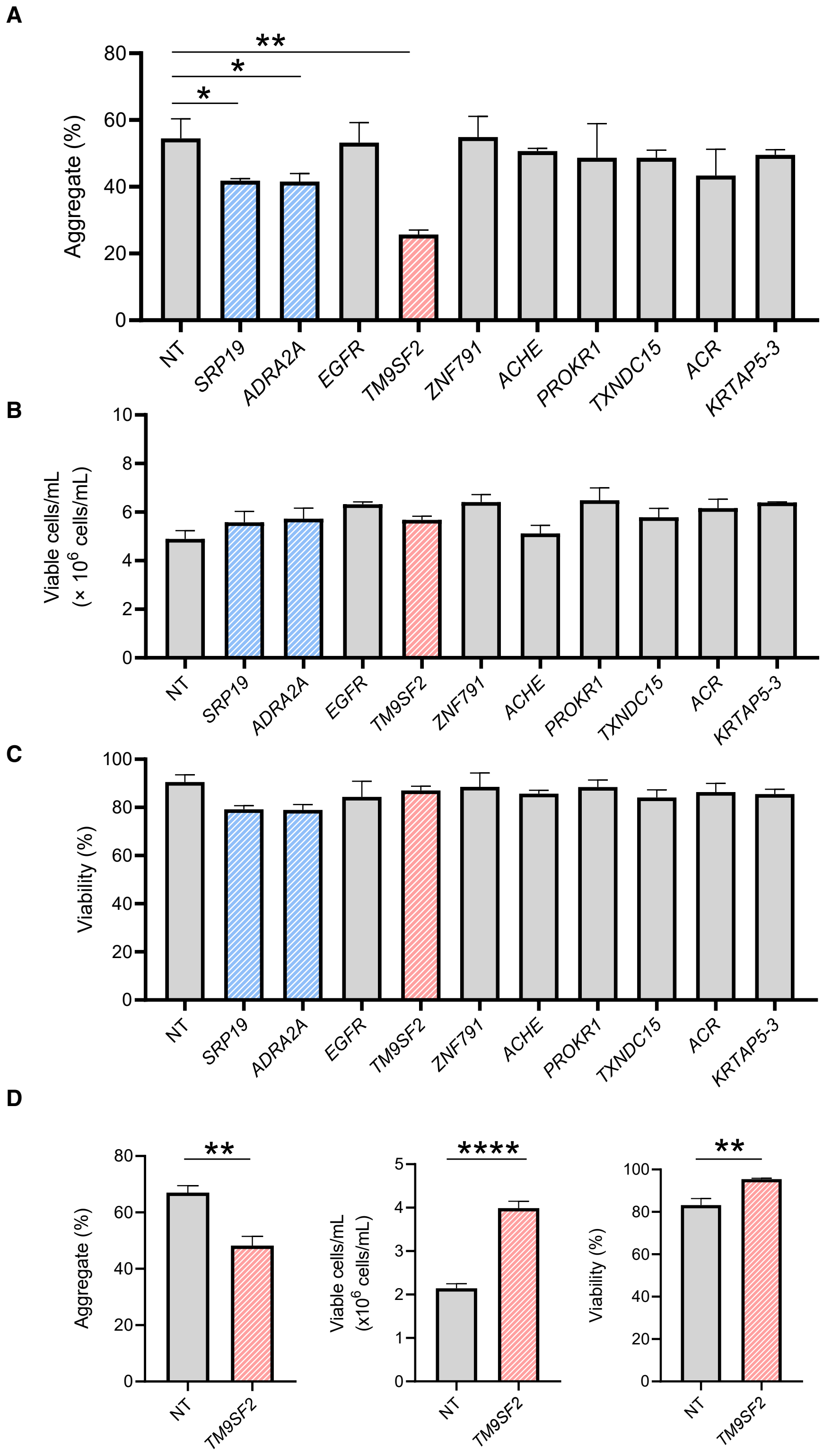

Figure 5. Experimental validation of the top 10 hits
(A) Ratio of aggregated cells in the total cell count, (B) VCC, and (C) viability of non-targeting HEK293T control (NT) and HEK293T KO cell pools of the top 10 candidate genes. (D) Ratio of aggregated cells in the total cell count, VCC, and viability of non-targeting HEK293 control (NT) and HEK293 *TM9SF2* KO cell pool. Cells were seeded at a concentration of 1.0×10^6 cells/mL in 6-well plates with 3 mL culture volume. After 3 days, cells were harvested undisturbed and analyzed by Cedex HiRES analyzer. Error bars represent the standard deviation calculated from three independent experiments. "ns" indicates nonsignificant value. $^*p < 0.05$, $^{**}p < 0.01$, $^{****}p < 0.0001$.

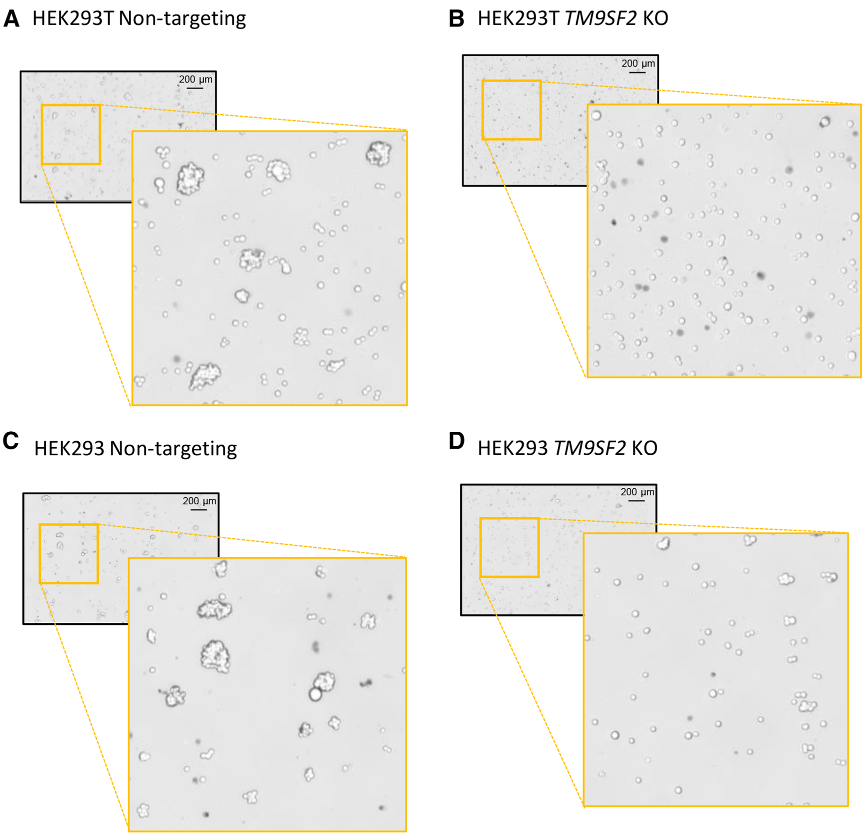

Figure 6. Microscope images showing decreased aggregation in TM9SF2 KO HEK293T and HEK293 cell pools compared with the NT controls

Images of (A) non-targeting HEK293T control, (B) *TM9SF2* KO HEK293T cell pool, (C) non-targeting HEK293 control, and (D) *TM9SF2* KO HEK293 cell pool. Bordered images depict enlarged portions in each part of the image. Cells were seeded at a concentration of 1.0 × 10^6 cells/mL in 6-well plates with 3 mL culture volume. After 3 days, cells were harvested undisturbed and analyzed by Cedex HiRES analyzer. Scale bars in all microscopic images are 200 μm.

that can reduce cell aggregation without adversely affecting cell growth.

Our screening revealed *TM9SF2*, *SRP19*, and *ADRA2A* as key targets whose KO effectively reduced cell aggregation after three days of suspension culture. Among the validated candidates, *TM9SF2* KO showed the most pronounced reduction in cell aggregation, with reductions of 53% and 28% compared with the NT control in HEK293T and HEK293 cells, respectively. This result motivated its selection as the primary target for further mechanistic investigation. *TM9SF2* encodes a transmembrane protein that is speculated to be involved in small molecule transport and ion channel regulation.[26] Importantly, *TM9SF2* has been shown to be required for the expression of bifunctional heparan sulfate N-deacetylase/N-sulfotransferase 1 (NDST1), a key enzyme in heparan sulfate (HS) biosynthesis.[27–30] Since HS plays a critical role in mediating cell-cell and cell-matrix interactions, it is plausible that the reduction in aggregation upon *TM9SF2* KO is due to altered HS synthesis and, consequently, modulated cell adhesion properties.

SRP19, another identified target, is essential for the early assembly of the signal recognition particle (SRP) and works in concert with SRP54 to ensure proper targeting of nascent proteins to the endoplasmic reticulum.[31] Although a role of *SRP19* in cell adhesion has not been previously reported, its function in co-translational protein targeting to the endoplasmic reticulum may suggest that disruption of *SRP19* could potentially influence the processing or localization of proteins involved in cell-cell interactions. However, the precise mechanism underlying the aggregation phenotype observed in this study remains unclear.

ADRA2A encodes the alpha-2A adrenergic receptor, which modulates intracellular signaling cascades that influence cell adhesion. *ADRA2A* primarily couples with Gi/o proteins, leading to inhibition of adenylate cyclase, reduced cyclic AMP (cAMP) levels, and downstream effects on intracellular calcium signaling.[11] KO of *ADRA2A* may disrupt these signaling pathways, thereby altering calcium homeostasis and influencing adhesion molecule expression or function.[11,32,33] This disruption could contribute to the observed decrease in cell aggregation.

Among the genetic targets identified through the CRISPR-Cas9 screen, *TM9SF2* KO was confirmed to have the most pronounced effect in reducing cell aggregation without compromising cell viability. Notably, the enriched GO terms across the BP, CC, and MF categories were largely associated with biological processes and cellular components related to cell-cell interactions. These enrichment patterns may suggest that *TM9SF2* could be involved in pathways regulating cell adhesion and coordinated cellular behavior, although the precise biological role of *TM9SF2* remains to be further elucidated. Integrated analysis of CRISPR screening data and RNA-seq profiling of *TM9SF2* KO cells revealed enrichment of pathways associated with the cell membrane, ECM, cytoskeletal organization, calcium signaling, and key adhesion-related pathways such as MAPK, Wnt, and PI3K-Akt. These pathways are well-established regulators of cell-cell and cell-matrix interactions, supporting the biological relevance of the screening results.[11–20] The concurrent enrichment of MAPK signaling, calcium-related processes, and membrane-associated terms across both datasets also indicates that these processes may be involved in the regulation of cell aggregation. Collectively, these findings suggest that *TM9SF2* may play a role in regulating cell aggregation in HEK293 cells and highlight potential strategies

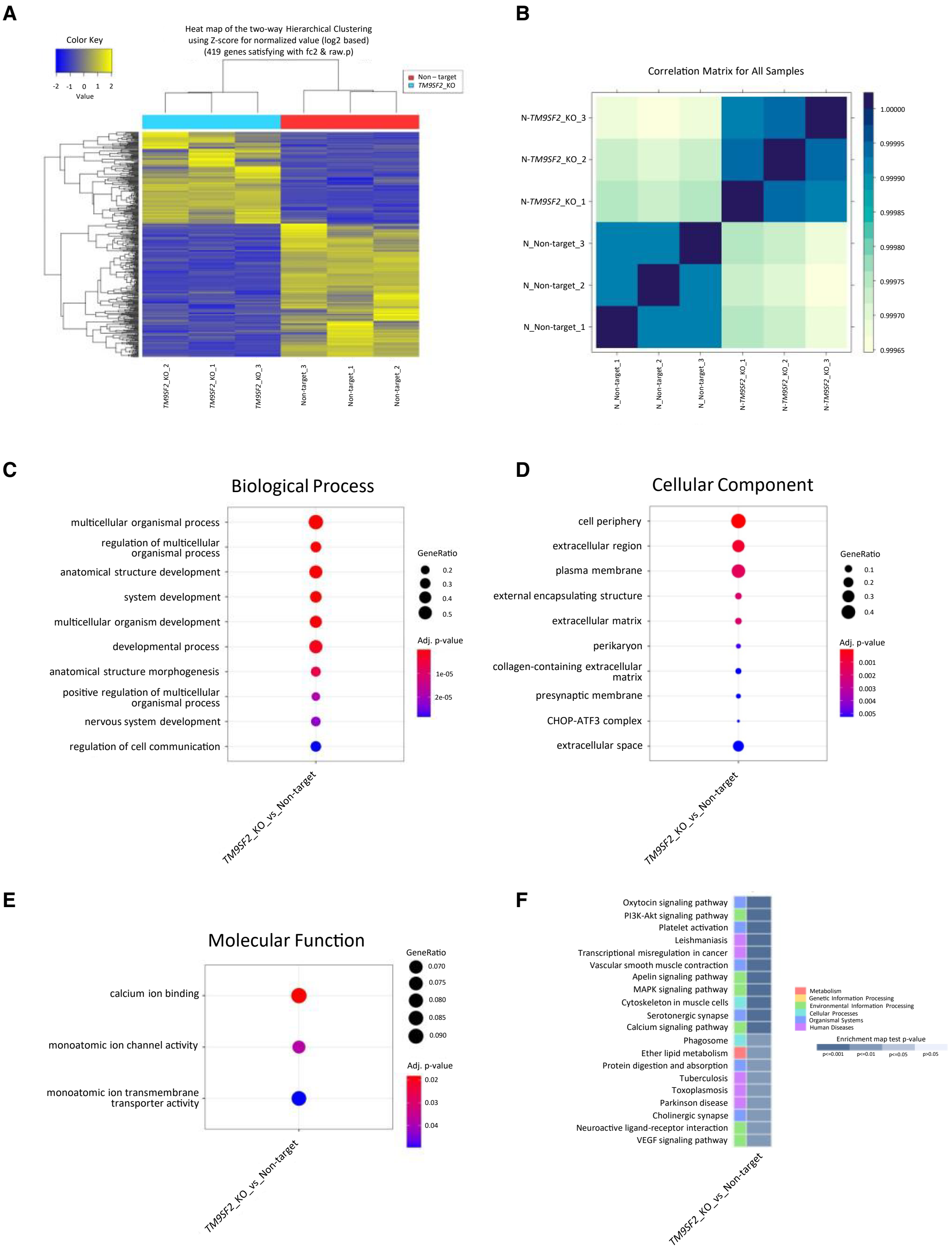

Figure 7. RNA-seq analysis of *TM9SF2* KO HEK293T cell pool compared with non-targeting HEK293T cell pool
(A) Heatmap of the two-way hierarchical clustering using Z score for normalized value with 419 genes satisfying $|fc| \geq 2$ and p value <0.05. (B) Correlation matrix for all samples. Functional GO term of enriched genes. Dot plot of the top 10 enriched (C) biological process (BP), (D) cellular component (CC), and top 3 (E) molecular function from

(legend continued on next page)

for engineering cell lines with reduced aggregation to improve performance in suspension culture and large-scale AAV production.

Although *TM9SF2* KO reduced cell aggregation, this did not lead to increased AAV titers under our experimental conditions, likely because the resuspension step prior to transfection partially disrupted pre-existing aggregates and minimized differences between NT and *TM9SF2* KO cells at the time of transfection. Nevertheless, in industrially relevant HEK293 suspension platforms for AAV or transient gene expression production, cell aggregation remains a recognized process-related challenge and may compromise culture performance, including cell growth and transfection efficiency.[21,23,24,34,35] Moreover, nutrient feeding at high cell densities can further exacerbate aggregation, negatively affecting overall culture performance and AAV production. Therefore, the aggregation-reducing phenotype of *TM9SF2* KO may provide greater advantages under industrial high-density culture conditions used for large-scale AAV manufacturing. Regardless of AAV serotypes tested, *TM9SF2* KO did not significantly affect genome titer. Furthermore, except for cell-associated AAV2, *TM9SF2* KO did not significantly affect full capsid ratio as well as infectivity of all secreted AAV serotypes (AAV5, AAV8, and AAV9). These properties are critical quality attributes (CQAs) in AAV production, as they directly impact drug efficacy and are difficult or costly to improve during downstream processing. Therefore, the lack of negative impact by *TM9SF2* KO on these CQAs makes it a highly attractive option for AAV manufacturing.

It is noteworthy that *TM9SF2* KO reduced the infectivity of AAV2. Given that *TM9SF2* has been implicated in heparan sulfate (HS) biosynthesis[27–30] and that AAV2 relies on heparan sulfate proteoglycan (HSPG) as its primary cellular receptor,[36,37] alterations in HS-related pathways may influence AAV2 capsid infectivity during particle assembly or maturation. However, this explanation remains speculative, and the precise mechanism requires further investigation.

Furthermore, in the present study, we focused on commonly used AAV production metrics, including VG titer, capsid titer, and infectious titer. However, further structural and biochemical characterization of vectors produced from *TM9SF2* KO cells will be important to fully understand potential related effects.

From a manufacturing standpoint, the extracellular release of AAV vectors offers significant advantages by simplifying downstream processing and minimizing the need for cell lysis and clarification. Despite the observed reduction in intracellular AAV2 infectivity following *TM9SF2* KO, the possibility of enhancing the genome titers of extracellular AAV5, AAV8, and AAV9, without adversely affecting full capsid ratios or infectivity, positions *TM9SF2* KO as an attractive strategy for large-scale production. Given that many commonly used AAV serotypes are predominantly secreted into the culture medium, the *TM9SF2* KO phenotype may be compatible with these production characteristics and could potentially be advantageous for suspension-based AAV manufacturing processes.

Moreover, the application of *TM9SF2* KO may extend beyond HEK293 cells to other cell lines that have traditionally been challenging to adapt to suspension culture. For instance, while MDCK cells are widely utilized for influenza vaccine production, their anchorage dependence limits scalability. Although suspension growth of MDCK cells has been achieved through transfection with the human *siat7e* gene,[38] *TM9SF2* KO could potentially further facilitate or optimize this adaptation.

In conclusion, using a virus-free HEK293T CRISPR-Cas9 KO library, *TM9SF2* was identified as a key target for overcoming the persistent challenge of cell aggregation in HEK293-based systems. Given the increasing demand for robust, high-yield HEK293 platforms for AAV manufacturing, this finding provides valuable insight and offers a promising approach to optimize upstream processes, thereby advancing scalable production of AAV vectors.

MATERIALS AND METHODS

Plasmids

For MCL development, *ROSA26*-LP donor and pU6-(*ROSA26*) CBh-Cas9-T2A-BFP were used. Annealed *ROSA26*-targeting gRNAs were cloned into pU6-(BbsI) CBh-Cas9-T2A-BFP vector (Addgene plasmid # 64323, a kind gift from Ralf Kuehn) for cloning pU6-(*ROSA26*) CBh-Cas9-T2A-BFP vector, as previously described.[39] For the RMCE efficiency test, PuroR-attB-Esp3I and NP-C-NLS-Bxb1 were used. For the development of the cell-based gRNA library, gRNA library plasmids, which were cloned for RMCE and NP-C-NLS-Bxb1,[40] were used. For the KO efficiency test, gRNA(*BAK1*)-Cas9-T2A-Blast was used. For KO library cell pool development, the Cas9-Blast vector was used. For KO validation, annealed NT and targeting gRNAs were cloned into pSpCas9(BB)-T2A-Hygro vector (Addgene plasmid #118055, a kind gift from Ken-Ichi Takemaru), as previously described.[39] All the plasmids used in this study are summarized in Table S2.

For AAV production, pHelper (a kind gift from Stratagene), pAAV2/2 (Addgene plasmid #104963, a kind gift from Melina Fan), pAAV2/5 (Addgene plasmid #104964, a kind gift from Melina Fan), pAAV2/8 (Addgene plasmid #112864, a kind gift from James M.Wilson), pAAV2/9n (Addgene plasmid #128865, a kind gift from James M.Wilson), and pAAV-GOI (Addgene plasmid #105530, a kind gift from James M.Wilson) were used. All the primers and sgRNA oligos used in this study are summarized in Tables S3 and S4, respectively.

GO enrichment analysis, ranked by adjusted *p* value for *TM9SF2* KO HEK293T cell pool compared with non-targeting HEK293T control. (F) KEGG gene set enrichment analysis results of the top 20 pathways ranked by *p* value. Cells were seeded at a concentration of 1.0×10^6 cells/mL in 6-well plates with 3 mL culture volume. After 3 days, cells from each group were harvested, RNA was extracted, and transcriptome sequencing was performed using the NovaSeqX platform. Sequencing reads were aligned to the human genome reference (GRCh38) to analyze the transcriptomic impact of *TM9SF2* KO HEK293T cells compared with non-targeting HEK293T control.

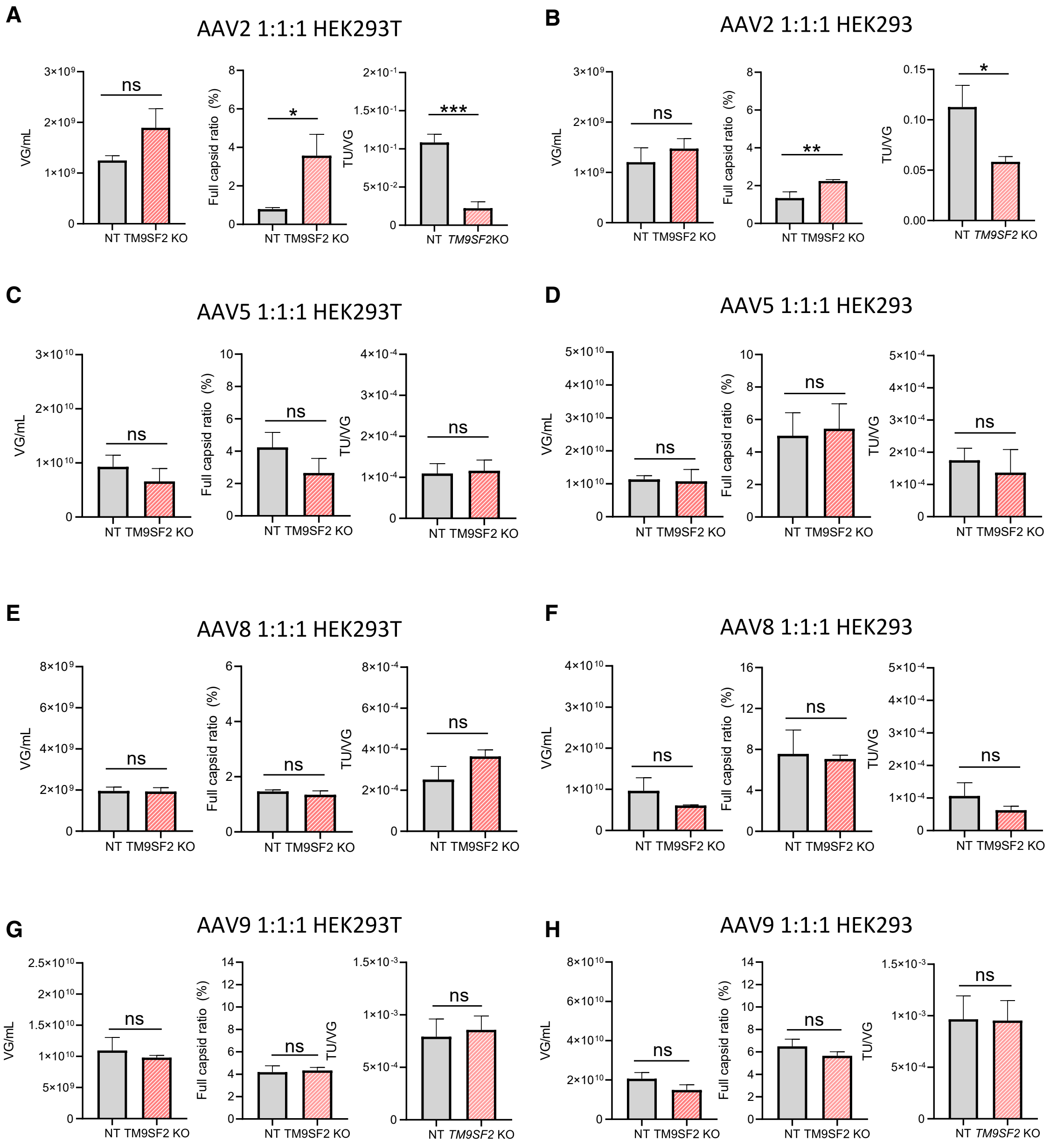

Figure 8. Investigation of the impact of *TM9SF2* KO on AAV2, AAV5, AAV8, and AAV9 production

(A) Assessment of genome titer, full capsid ratio, and TU/VG during AAV2 production in HEK293T cells. (B) Assessment of genome titer, full capsid ratio, and TU/VG during AAV2 production in HEK293 cells. (C) Assessment of genome titer, full capsid ratio, and TU/VG during AAV5 production in HEK293T cells. (D) Assessment of genome titer, full capsid ratio, and TU/VG during AAV5 production in HEK293 cells. (E) Assessment of genome titer, full capsid ratio, and TU/VG during AAV8 production in HEK293T cells. (F) Assessment of genome titer, full capsid ratio, and TU/VG during AAV8 production in HEK293 cells. (G) Assessment of genome titer, full capsid ratio, and TU/VG during AAV9 production in HEK293T cells. (H) Assessment of genome titer, full capsid ratio, and TU/VG during AAV9 production in HEK293 cells. Intracellular AAV2 and extracellular AAV5, AAV8, and AAV9 were produced as previously described.[1,4] Error bars represent the standard deviation calculated from three independent experiments. "ns" indicates nonsignificant value. $^{*}p < 0.05$, $^{**}p < 0.01$, $^{***}p < 0.001$.

Library construction

The Human Brunello CRISPR KO pooled library (Addgene #73178, a kind gift from David Root and John Doench) was PCR-amplified and cloned into the PuroR-attB-Esp3I backbone using the Gibson Assembly Master Mix (New England Biolabs, Ipswich, MA), as previously described.[39] The resulting plasmid library was transformed into Endura ElectroCompetent Cells (Lucigen, Middleton, WI) with an electroporation efficiency of 500 copies per gRNA per library using a MicroPulser Electroporator (Bio-Rad, Hercules, CA). Transformed cells were grown overnight in Lysogeny Broth (LB) with

ampicillin at 37°C, and plasmids were then isolated using the NucleoBond Xtra Maxi EF Kit (Macherey-Nagel).

Cell culture and transfection

HEK293T cells (ATCC number: CRL-3216) were maintained by seeding at a concentration of 3 × 10^5 cells/mL in fresh medium every 3–4 days in 125 mL Erlenmeyer flasks (Corning, Corning, NY) containing 30 mL of FreeStyle F17 expression medium (Gibco, Grand Island, NY) supplemented with 4 mM glutamine (Gibco). Cells were incubated at 37°C with 5% (v/v) CO_2 and shaken at 110 rpm in a climo-shaking CO_2 incubator (Adolf Kuhner AG, Birsfelden, Switzerland) as previously described.[1,4] To ensure the coverage of 500 cells per gRNA, gRNA cell pool and KO library cell pool were maintained by seeding at a concentration of 5 × 10^5 cells/mL every 3–4 days in two 125 mL Erlenmeyer flasks (Corning) containing 50 mL of FreeStyle F17 expression medium (Gibco, Grand Island, NY) supplemented with 4 mM glutamine. For transfection, cells were seeded at a concentration of 1 × 10^6 cells/mL in fresh medium and transfected with PEIMAX (Polysciences, Warrington, PA), as previously described.[1,4]

Development of the HEK293T cell-based SLP MCL using the CRISPR-Cas9-mediated RMCE landing pad system

HEK293T SLP MCL was generated as previously described.[39,41] HEK293T cells were seeded at a concentration of 1 × 10^6 cells/mL in 125 mL Erlenmeyer flasks containing 30 mL culture media. Subsequently, cells were transfected with an *ROSA26*-LP donor plasmid and pU6-(*ROSA26*) CBh-Cas9-T2A-BFP plasmid at 1:1 weight ratio using PEIMAX, as previously described.[1,4] Cells were treated with 100 μg/mL hygromycin (Invivogen, San Diego, CA) for 3 days, followed by recovery for 4 passages without hygromycin, as previously described.[39] Then, cells were single-cell sorted in 96 well plates using Moflo Astrios EQ (Beckman Coulter, Brea, CA). The clones were expanded, and the landing pad was verified using 5′/3′ junction PCR. Validation of a single copy of the landing pad was confirmed by RT-qPCR using a reference cell line, as previously described.[39,41] Out-out PCR was conducted for final confirmation of full insertion of landing pad.

Development of a HEK293T cell-based gRNA library

To ensure the coverage of 500 cells per gRNA, which can guarantee representation of the gRNA library, the number of cells required for transfection was calculated based on the measured value of RMCE efficiency on day 11 after transfection, which was 3.3% (Figure S2). A total of 1.2 × 10^9 cells were seeded at a concentration of 1.0 × 10^6 cells/mL in twenty-four 125 mL Erlenmeyer flasks (Corning), each containing 50 mL of FreeStyle F17 expression medium (Gibco) supplemented with 4 mM glutamine. Subsequently, cells were transfected with gRNA library plasmid and NP-C-NLS-Bxb1 plasmid at a 3:1 weight ratio[40] using PEIMAX, as previously described.[1,4] 48 hours post transfection (HPT), cells were passaged every 3 days with 2 μg/mL puromycin. 17 days after transfection, recovered cell pools were subjected to Cas9 transfection, and a total of 4.0 × 10^7 cells were used for genomic DNA extraction.

Establishment of a knockout library cell pool

To ensure sufficient coverage, a total of 1.0 × 10^8 cells were seeded at a concentration of 1.0 × 10^6 cells/mL in two 125 mL Erlenmeyer flasks (Corning), each containing 50 mL of FreeStyle F17 expression medium (Gibco) with 4 mM glutamine. Subsequently, cells were transfected with Cas9-Blast plasmid[41] using PEIMAX, as previously described.[1,4] At 48 HPT, a total of 2.0 × 10^8 cells were treated with 400 μg/mL blasticidin (Sigma) for 3 days, followed by recovery for 4 passages without blasticidin (Sigma). Blasticidin concentration was determined based on KO efficiency test with gRNA(*BAK1*)-Cas9-T2A-BSD vector and HEK293T cell-based gRNA library (Figure S3). Recovered KO library cell pool was subjected to aggregation screening, and a total of 4.0 × 10^7 cells were used for genomic DNA extraction.

Enrichment of non-aggregated cell population in KO library cell pool

To enrich non-aggregated cell population, KO library cells were initially seeded at a density of 1.0 × 10^6 cells/mL in 125 mL Erlenmeyer flasks containing 50 mL of culture medium. After two days of cultivation, cell suspensions from the flasks (48 mL) were transferred into 50 mL conical tubes (Corning) and allowed to stand undisturbed for 9 min to permit sedimentation of cell aggregates. Subsequently, 24 mL of the upper (non-aggregated) cell suspension was carefully collected. Of this, 0.5 mL was used to determine VCC, while the remaining 23.5 mL was centrifuged and resuspended in 50 mL of fresh culture medium in new Erlenmeyer flasks. This separation and enrichment procedure was repeated every three days to progressively enrich for non-aggregated cells, until phenotypic differences between the control and experimental groups became evident. For the control group, cells were passaged every three days at the same initial density of 1.0 × 10^6 cells/mL in 125 mL Erlenmeyer flasks with 50 mL of culture medium without performing the cell separation step.

Sample preparation for NGS

Genomic DNA samples were extracted using the Exgene Blood SV kit (GeneAll, Seoul, South Korea), according to the manufacturer's protocol. To prepare NGS samples, PCR was performed using primers listed in Table S3, as previously described.[41] The PCR products were purified using a Expin Combo GP (GeneAll) and indexed using a TruSeq Nano DNA Library Prep Kit (Illumina, San Diego, CA). The indexed library was measured using qPCR according to the Illumina qPCR Quantification Protocol Guide (Illumina). The library size was assessed with a TapeStation D1000 ScreenTape (Agilent Technologies, Santa Clara, CA) and sequenced on a NextSeq500 sequencer (Illumina).

NGS and data analysis

For the analysis of gRNA fold changes during screening, raw FASTQ files, along with files containing all gRNA sequences for each library, were processed using PinAPL-Py[22] with default settings. Non-aggregated cells after four rounds of selection were compared with the control to identify gRNAs that were either enriched or depleted

across three replicates. These gRNAs and target genes were ranked using αRRA algorithm. To control the false discovery rate (FDR), *p* values were adjusted using the Benjamini-Hochberg correction method. PPI analysis was performed using the STRING database (https://string-db.org) with the list of 93 genes identified from the screening (≥2 sgRNAs per gene). STRING integrates multiple sources of evidence, including experimentally validated interactions, curated databases, co-expression, gene neighborhood, gene fusion events, and text mining, to construct a functional interaction network.

Generation and validation of KO pools

To establish a KO suspension cell pool, HEK293T cells were seeded at a concentration of 1×10^6 cells/mL in a 6-well plate containing FreeStyle F17 expression medium (Gibco) supplemented with 4 mM glutamine and transfected with all-in-one pSpCas9(BB)-T2A-Hygro vectors engineered to target specific genes, as previously described.[4] At 48 HPT, the transfected cells were treated with 100 μg/mL hygromycin for 3 days, followed by 3–4 passages of recovery without hygromycin. A control suspension cell pool was generated in parallel by transfecting HEK293T suspension cells with an NT version of the vector using the same procedure. After recovery, gene KO was validated using TIDE analysis.[42]

Validation of enhancement in cell aggregation

For validation of enhancement in cell aggregation of top 10 hits, target gene KO cell pools were seeded at a concentration of 1.0×10^6 cells/mL and cultured for 3 days in 6-well plate containing 3 mL FreeStyle F17 expression medium (Gibco) supplemented with 4 mM glutamine (Gibco) in a climo-shaking CO_2 incubator. An NT control cell pool was cultured in parallel. Cell aggregation, VCC, and viability were measured and cells were pictured via Cedex HiRes analyzer (Roche, Bazel, Switzerland).

Transcriptome re-sequencing

NT HEK293T cells and *TM9SF2* KO HEK293T cell pool were seeded at a concentration of 1.0×10^6 cells/mL and cultured for 3 days in 6-well plate containing 3 mL FreeStyle F17 expression medium (Gibco) supplemented with 4 mM glutamine. After 3 days, cells were collected from each group, and RNA was extracted for RNA-seq analysis. Sequencing was performed on the Illumina NovaSeqX platform, and alignment was based on the *Homo sapiens* GRCh38 reference genome (NCBI release 109.20200522). Raw data obtained from RNA-seq were pre-processed using Trimmomatic to remove low-quality reads and adapter sequences, resulting in high-quality trimmed reads. These trimmed reads were then aligned to the reference genome using HISAT2, a spliced alignment tool, allowing for precise mapping. Transcript assembly and quantification of gene expression levels were performed using StringTie. Expression was measured as read counts, FPKM (fragments per kilobase of transcript per million mapped reads), and TPM (transcripts per kilobase million). Differential expression analysis was conducted using DESeq2 to compare the NT HEK293T cells and *TM9SF2* KO HEK293T cell pool. Genes with $|\text{fold change (fc)}| \geq 2$ and p value <0.05 were considered significantly differentially expressed. Detailed results of analyses are provided in Tables S5, S6, S7, S8, and S9.

AAV production

AAV2, AAV5, AAV8, and AAV9 were produced as previously described.[1,4] Cells were seeded at a concentration of 1.0×10^6 cells/mL in 6-well plates (SPL, Pocheon, Korea) containing 3 mL FreeStyle F17 expression medium (Gibco) supplemented with 4 mM glutamine. Transfection was performed using transfection cocktails containing plasmids at 1:1:1 weight ratios (pHelper:-pAAV2/2 or pAAV2/9n:pAAV-GOI).[1,4] The transfection cocktails were prepared by adding 13.5 μg of PEIMAX to the plasmid mixture, which contained a total of 4.5 μg of plasmids in 150 μL of Opti-MEM. AAV2 was harvested at 48 HPT in cell lysate, while AAV5, AAV8, and AAV9 were harvested at 144 HPT in cell culture media, as previously described.[1,4]

AAV recovery

AAV recovery was conducted as previously described.[1,4] To recover AAV2, transfected cells were centrifuged at 2000 ×*g* for 20 min, resuspended in AAV lysis buffer (150 mM NaCl, 50 mM Tris, pH 8.4), and subjected to three freeze-thaw cycles. Lysates were treated with Benzonase (10 U/mL) for 30 min at 37°C, centrifuged at 1600 ×*g* for 5 min at room temperature and then at 16,600 ×*g* for 10 min at 4°C. Supernatants were stored at −80°C. To recover AAV5, AAV8, and AAV9, cells were removed via centrifugation, and the culture supernatant was mixed with AAV precipitation solution (2.5 M NaCl, 40% polyethylene glycol 8000 in distilled water) at a 1:4 volume ratio and incubated on ice at 4°C for 2 h. The AAV precipitate was centrifuged at 16,000 ×*g* for 10 min at 4°C, resuspended in AAV lysis buffer with 10% sodium deoxycholate at a 1:20 volume ratio, treated with Benzonase at 37°C for 30 min, and stored at −80°C.

AAV titration

AAV genome titer was measured as previously described.[1,4] For sample pre-treatment, serially diluted AAV samples were treated with DNase I at 37°C for 30 min, followed by stop solution for 90 s at room temperature, and then heated to 65°C for 10 min. Samples were then treated with 0.5 μL of Proteinase K for 90 s at room temperature, incubated at 50°C for 1 h, and at 95°C for 20 min. For AAV titration, RT-qPCR was performed with iQ SYBR Green Supermix using a CFX96 Real-Time System (Biorad, Hercules, CA). A standard curve was generated for each RT-qPCR run using pAAV-GOI, with concentrations ranging from 10^8 to 10^2 copies/μL, and primers against the CMV promoter. AAV capsid titers were measured using the PROGEN AAV titration ELISA kits (PROGEN, Heidelberg, Germany), according to the manufacturer's protocol. The full capsid ratio (%) was calculated by dividing genome titers measured by qPCR by capsid titers determined by ELISA. AAV functional titers were measured as previously described.[1,4] Adherent HEK293T cells were seeded in 48-well plates at a concentration of 0.5×10^6 cells/mL and cultured for 24 h prior to transduction. For transduction, the culture medium (DMEM containing 10% FBS) was removed and

100 μL of AAV diluted in fresh DMEM containing 10% FBS was added to each well. Viral dilutions were empirically optimized to achieve a transduction efficiency of approximately 2%–20%, thereby avoiding underestimation at high infection levels or overestimation at very low infection levels. Cells were incubated with the virus at 37°C for 1 h, after which 400 μL of DMEM containing 10% FBS was added to each well. Cells were further cultured for 48 h, harvested, and analyzed for transduction efficiency. All experiments were performed with three biological replicates ($n = 3$).

Statistical analysis

Unless otherwise specified, data are presented as mean ± standard error of the mean (SEM), $n = 3$. Statistical significance was calculated using an unpaired Student's t test; it was set at $p < 0.05$.

DATA AND CODE AVAILABILITY

Data are available on request from the authors.

ACKNOWLEDGMENTS

This research was supported by Merck Life Science. We would like to thank Hoon-Min Lee and Yeon-Gu Kim from Biotherapeutics Translational Research Center in Korea Research Institute of Bioscience and Biotechnology (KRIBB) for technical assistance with the Cedex HiRES analyzer. This work was supported by Merck Life Science and by the Technology Innovation Program (RS-2024-00408022), funded by the Ministry of Trade, Industry and Energy, Republic of Korea.

AUTHOR CONTRIBUTIONS

Conceptualization, S.P. and G.M.L.; methodology, S.P., S.S, G.H., Y.W., and S.Y.L.; validation, S.P., G.H., and Y.W.; formal analysis, S.P., S.S., G.H., Y.W., and S.Y.L.; investigation, S.P., G.H., and Y.W.; resources, D.R. and H.G.; data curation, S.P. and S.S.; writing, S.P., Y.W., D.R., H.G., and G.M.L.; supervision, S.S. and G.M.L.; project administration, D.R., H.G., and G.M.L.; and funding acquisition, D.R., H.G., and G.M.L.

DECLARATION OF INTERESTS

The authors declare no competing interests.

SUPPLEMENTAL INFORMATION

Supplemental information can be found online at https://doi.org/10.1016/j.omta.2026.201777.

REFERENCES

1. Park, S., Shin, S., Lee, H., Jang, J.H., and Lee, G.M. (2024). Enhancing the production of adeno-associated virus (AAV)2 and AAV9 with high full capsid ratio in HEK293 cells through design-of-experiment optimization of triple plasmid ratio. Biotechnol. J. *19*, e2300667. https://doi.org/10.1002/biot.202300667.
2. Ayuso, E., Mingozzi, F., and Bosch, F. (2010). Production, purification and characterization of adeno-associated vectors. Curr. Gene Ther. *10*, 423–436. https://doi.org/10.2174/156652310793797685.
3. Robert, M.A., Chahal, P.S., Audy, A., Kamen, A., Gilbert, R., and Gaillet, B. (2017). Manufacturing of recombinant adeno-associated viruses using mammalian expression platforms. Biotechnol. J. *12*, e1600193. https://doi.org/10.1002/biot.201600193.
4. Park, S., Shin, S., Han, G., and Lee, G.M. (2025). Knockout of pro-apoptotic BAX and BAK1 genes in HEK293T cells enhances adeno-associated virus (AAV) production: AAV2 and AAV9. Biotechnol. J. *20*, e202400529. https://doi.org/10.1002/biot.202400529.
5. Chahal, P.S., Schulze, E., Tran, R., Montes, J., and Kamen, A.A. (2014). Production of adeno-associated virus (AAV) serotypes by transient transfection of HEK293 cell suspension cultures for gene delivery. J. Virol. Methods *196*, 163–173. https://doi.org/10.1016/j.jviromet.2013.10.038.
6. Zhao, H., Lee, K.J., Daris, M., Lin, Y., Wolfe, T., Sheng, J., Plewa, C., Wang, S., and Meisen, W.H. (2020). Creation of a high-yield AAV vector production platform in suspension cells using a design-of-experiment approach. Mol. Ther. Methods Clin. Dev. *18*, 312–320. https://doi.org/10.1016/j.omtm.2020.06.004.
7. Grieger, J.C., Soltys, S.M., and Samulski, R.J. (2016). Production of recombinant adeno-associated virus vectors using suspension HEK293 cells and continuous harvest of vector from the culture media for GMP FIX and FLT1 clinical vector. Mol. Ther. *24*, 287–297. https://doi.org/10.1038/mt.2015.187.
8. de los Milagros Bassani Molinas, M., Beer, C., Hesse, F., Wirth, M., and Wagner, R. (2013). Optimizing the transient transfection process of HEK-293 suspension cells for protein production by nucleotide ratio monitoring. Cytotechnology *66*, 493–514. https://doi.org/10.1007/s10616-013-9601-3.
9. Hyoung Park, J., Sin Lim, M., Rang Woo, J., Won Kim, J., and Min Lee, G. (2016). The molecular weight and concentration of dextran sulfate affect cell growth and antibody production in CHO cell cultures. Biotechnol. Prog. *32*, 1113–1122. https://doi.org/10.1002/btpr.2287.
10. Elshereef, A.A., Jochums, A., Lavrentieva, A., Stuckenberg, L., Scheper, T., and Solle, D. (2019). High cell density transient transfection of CHO cells for TGF-β1 expression. Eng. Life Sci. *19*, 730–740. https://doi.org/10.1002/elsc.201800174.
11. Alberts, B., Heald, R., Johnson, A., Morgan, D., Raff, M.C., Roberts, K., and Walter, P. (2022). Molecular Biology of the Cell, 7th edition (W. W. Norton & Company).
12. Kato, H., Gruenwald, A., Suh, J.H., Miner, J.H., Barisoni-Thomas, L., Taketo, M.M., Faul, C., Millar, S.E., Holzman, L.B., and Susztak, K. (2011). Wnt/β-catenin pathway in podocytes integrates cell adhesion, differentiation, and survival. J. Biol. Chem. *286*, 26003–26015. https://doi.org/10.1074/jbc.M111.223164.
13. Pece, S., and Gutkind, J.S. (2000). Signaling from E-cadherins to the MAPK pathway by the recruitment and activation of epidermal growth factor receptors upon cell-cell contact formation. J. Biol. Chem. *275*, 41227–41233. https://doi.org/10.1074/jbc.M006578200.
14. Schaefer, A.W., Kamiguchi, H., Wong, E.V., Beach, C.M., Landreth, G., and Lemmon, V. (1999). Activation of the MAPK signal cascade by the neural cell adhesion molecule L1 requires L1 internalization. J. Biol. Chem. *274*, 37965–37973. https://doi.org/10.1074/jbc.274.53.37965.
15. Matsuoka, T., Yashiro, M., Nishioka, N., Hirakawa, K., Olden, K., and Roberts, J.D. (2012). PI3K/Akt signalling is required for the attachment and spreading, and growth in vivo of metastatic scirrhous gastric carcinoma. Br. J. Cancer *106*, 1535–1542. https://doi.org/10.1038/bjc.2012.107.
16. Guidetti, G.F., Canobbio, I., and Torti, M. (2015). PI3K/Akt in platelet integrin signaling and implications in thrombosis. Adv. Biol. Regul. *59*, 36–52. https://doi.org/10.1016/j.jbior.2015.06.001.
17. Godoy-Parejo, C., Deng, C., Liu, W., and Chen, G. (2019). Insulin stimulates PI3K/AKT and cell adhesion to promote the survival of individualized human embryonic stem cells. Stem Cell *37*, 1030–1041. https://doi.org/10.1002/stem.3026.
18. Ihermann-Hella, A., Lume, M., Miinalainen, I.J., Pirttiniemi, A., Gui, Y., Peränen, J., Charron, J., Saarma, M., Costantini, F., and Kuure, S. (2014). Mitogen-activated protein kinase (MAPK) pathway regulates branching by remodeling epithelial cell adhesion. PLoS Genet. *10*, e1004193. https://doi.org/10.1371/journal.pgen.1004193.
19. Xu, J., Zhou, J., Dai, H., Liu, F., Li, W., Wang, W., and Guo, F. (2018). CHIP functions as an oncogene by promoting colorectal cancer metastasis via activation of MAPK and AKT signaling and suppression of E-cadherin. J. Transl. Med. *16*, 169. https://doi.org/10.1186/s12967-018-1540-5.
20. Ruan, Y., Chen, L., Xie, D., Luo, T., Xu, Y., Ye, T., Chen, X., Feng, X., and Wu, X. (2022). Mechanisms of cell adhesion molecules in endocrine-related cancers: A concise outlook. Front. Endocrinol. *13*, 865436. https://doi.org/10.3389/fendo.2022.865436.
21. Renner, W.A., Jordan, M., Eppenberger, H.M., and Leist, C. (1993). Cell-cell adhesion and aggregation: Influence on the growth behavior of CHO cells. Biotechnol. Bioeng. *41*, 188–193. https://doi.org/10.1002/bit.260410204.
22. Spahn, P.N., Bath, T., Weiss, R.J., Kim, J., Esko, J.D., Lewis, N.E., and Harismendy, O. (2017). PinAPL-Py: a comprehensive web-application for the analysis of CRISPR/Cas9 screens. Sci. Rep. *7*, 15854. https://doi.org/10.1038/s41598-017-16193-9.
23. Seidel, S., Maschke, R.W., Mozaffari, F., Eibl-Schindler, R., and Eibl, D. (2023). Improvement of HEK293 cell growth by adapting hydrodynamic stress and

predicting cell aggregate size distribution. Bioengineering *10*, 478. https://doi.org/10.3390/bioengineering10040478.

24. Faravelli, S., Campioni, M., Palamini, M., Canciani, A., Chiapparino, A., and Forneris, F. (2021). Optimized recombinant production of secreted proteins using human embryonic kidney (HEK293) cells grown in suspension. Bio Protoc. *11*, e3998. https://doi.org/10.21769/BioProtoc.3998.
25. Malm, M., Saghaleyni, R., Lundqvist, M., Giudici, M., Chotteau, V., Field, R., Varley, P.G., Hatton, D., Grassi, L., Svensson, T., et al. (2020). Evolution from adherent to suspension: systems biology of HEK293 cell line development. Sci. Rep. *10*, 18996. https://doi.org/10.1038/s41598-020-76137-8.
26. Clark, C.R., Maile, M., Blaney, P., Hellweg, S.R., Strauss, A., Durose, W., Priya, S., Habicht, J., Burns, M.B., Blekhman, R., et al. (2018). Transposon mutagenesis screen in mice identifies TM9SF2 as a novel colorectal cancer oncogene. Sci. Rep. *8*, 15327. https://doi.org/10.1038/s41598-018-33527-3.
27. Yamaji, T., Sekizuka, T., Tachida, Y., Sakuma, C., Morimoto, K., Kuroda, M., and Hanada, K. (2019). A CRISPR screen identifies LAPTM4A and TM9SF proteins as glycolipid-regulating factors. iScience *11*, 409–424. https://doi.org/10.1016/j.isci.2018.12.039.
28. Luteijn, R.D., van Diemen, F., Blomen, V.A., Boer, I.G.J., Manikam Sadasivam, S., van Kuppevelt, T.H., Drexler, I., Brummelkamp, T.R., Lebbink, R.J., and Wiertz, E.J. (2019). A genome-wide haploid genetic screen identifies heparan sulfate-associated genes and the macropinocytosis modulator TMED10 as factors supporting vaccinia virus infection. J. Virol. *93*, e02160-18. https://doi.org/10.1128/JVI.02160-18.
29. Meisen, W.H., Nejad, Z.B., Hardy, M., Zhao, H., Oliverio, O., Wang, S., Hale, C., Ollmann, M.M., and Collins, P.J. (2020). Pooled screens identify GPR108 and TM9SF2 as host cell factors critical for AAV transduction. Mol. Ther. Methods Clin. Dev. *17*, 601–611. https://doi.org/10.1016/j.omtm.2020.03.012.
30. Tanaka, A., Tumkosit, U., Nakamura, S., Motooka, D., Kishishita, N., Priengprom, T., Sa-ngasang, A., Kinoshita, T., Takeda, N., and Maeda, Y. (2017). Genome-wide screening uncovers the significance of N-sulfation of heparan sulfate as a host cell factor for Chikungunya virus infection. J. Virol. *91*, e00432-17. https://doi.org/10.1128/JVI.00432-17.
31. Hainzl, T., Huang, S., and Sauer-Eriksson, A.E. (2002). Structure of the SRP19-RNA complex and implications for signal recognition particle assembly. Nature *417*, 767–771. https://doi.org/10.1038/nature00768.
32. Clapham, D.E. (2007). Calcium signaling. Cell *131*, 1047–1058. https://doi.org/10.1016/j.cell.2007.11.028.
33. Sheng, L., Leshchyns'ka, I., and Sytnyk, V. (2013). Cell adhesion and intracellular calcium signaling in neurons. Cell Commun. Signal. *11*, 94. https://doi.org/10.1186/1478-811X-11-94.
34. Khalil, A.S., Yu, X., Dang, P.N., Alsberg, E., and Murphy, W.L. (2019). A microparticle approach for non-viral gene delivery within 3D human mesenchymal stromal cell aggregates. Acta Biomater. *95*, 408–417. https://doi.org/10.1016/j.actbio.2019.04.038.
35. Feng, L., Guo, M., Zhang, S., Chu, J., Zhuang, Y., and Zhang, S. (2007). Optimization of transfection mediated by calcium phosphate for plasmid rAAV-LacZ (recombinant adeno-associated virus-beta-galactosidase reporter gene) production in suspension-cultured HEK-293 (human embryonic kidney 293) cells. Biotechnol. Appl. Biochem. *46*, 127–135. https://doi.org/10.1042/BA20060143.
36. Opie, S.R., Warrington, K.H., Jr., Agbandje-McKenna, M., Zolotukhin, S., and Muzyczka, N. (2003). Identification of amino acid residues in the capsid proteins of adeno-associated virus type 2 that contribute to heparan sulfate proteoglycan binding. J. Virol. *77*, 6995–7006. https://doi.org/10.1128/jvi.77.12.6995-7006.2003.
37. Perabo, L., Goldnau, D., White, K., Endell, J., Boucas, J., Humme, S., Work, L.M., Janicki, H., Hallek, M., Baker, A.H., and Büning, H. (2006). Heparan sulfate proteoglycan binding properties of adeno-associated virus retargeting mutants and consequences for their in vivo tropism. J. Virol. *80*, 7265–7269. https://doi.org/10.1128/JVI.00076-06.
38. Chu, C., Lugovtsev, V., Golding, H., Betenbaugh, M., and Shiloach, J. (2009). Conversion of MDCK cell line to suspension culture by transfecting with human siat7e gene and its application for influenza virus production. Proc. Natl. Acad. Sci. USA *106*, 14802–14807. https://doi.org/10.1073/pnas.0905912106.
39. Shin, S., Kim, S.H., Park, J.H., Lee, J.S., and Lee, G.M. (2022). Recombinase-mediated cassette exchange-based screening of a CRISPR/Cas9 library for enhanced recombinant protein production in human embryonic kidney cells: Improving resistance to hyperosmotic stress. Metab. Eng. *72*, 247–258. https://doi.org/10.1016/j.ymben.2022.03.017.
40. Shin, S., Kim, S.H., Lee, J.S., and Lee, G.M. (2021). Streamlined human cell-based recombinase-mediated cassette exchange platform enables multigene expression for the production of therapeutic proteins. ACS Synth. Biol. *10*, 1715–1727. https://doi.org/10.1021/acssynbio.1c00113.
41. Kim, S.H., Shin, S., Baek, M., Xiong, K., Karottki, K.J.l.C., Hefzi, H., Grav, L.M., Pedersen, L.E., Kildegaard, H.F., Lewis, N.E., et al. (2023). Identification of hyperosmotic stress-responsive genes in Chinese hamster ovary cells via genome-wide virus-free CRISPR/Cas9 screening. Metab. Eng. *80*, 66–77. https://doi.org/10.1016/j.ymben.2023.09.006.
42. Brinkman, E.K., Chen, T., Amendola, M., and van Steensel, B. (2014). Easy quantitative assessment of genome editing by sequence trace decomposition. Nucleic Acids Res. *42*, e168. https://doi.org/10.1093/nar/gku936.

OMTA, Volume *34*

Supplemental information

CRISPR-Cas9 screening reveals *TM9SF2* knockout as a solution to HEK293 cell aggregation for improved AAV production

Sungje Park, Seunghyeon Shin, Gyucheol Han, Yubin Won, Sang Yoon Lee, David Razafsky, Henry George, and Gyun Min Lee

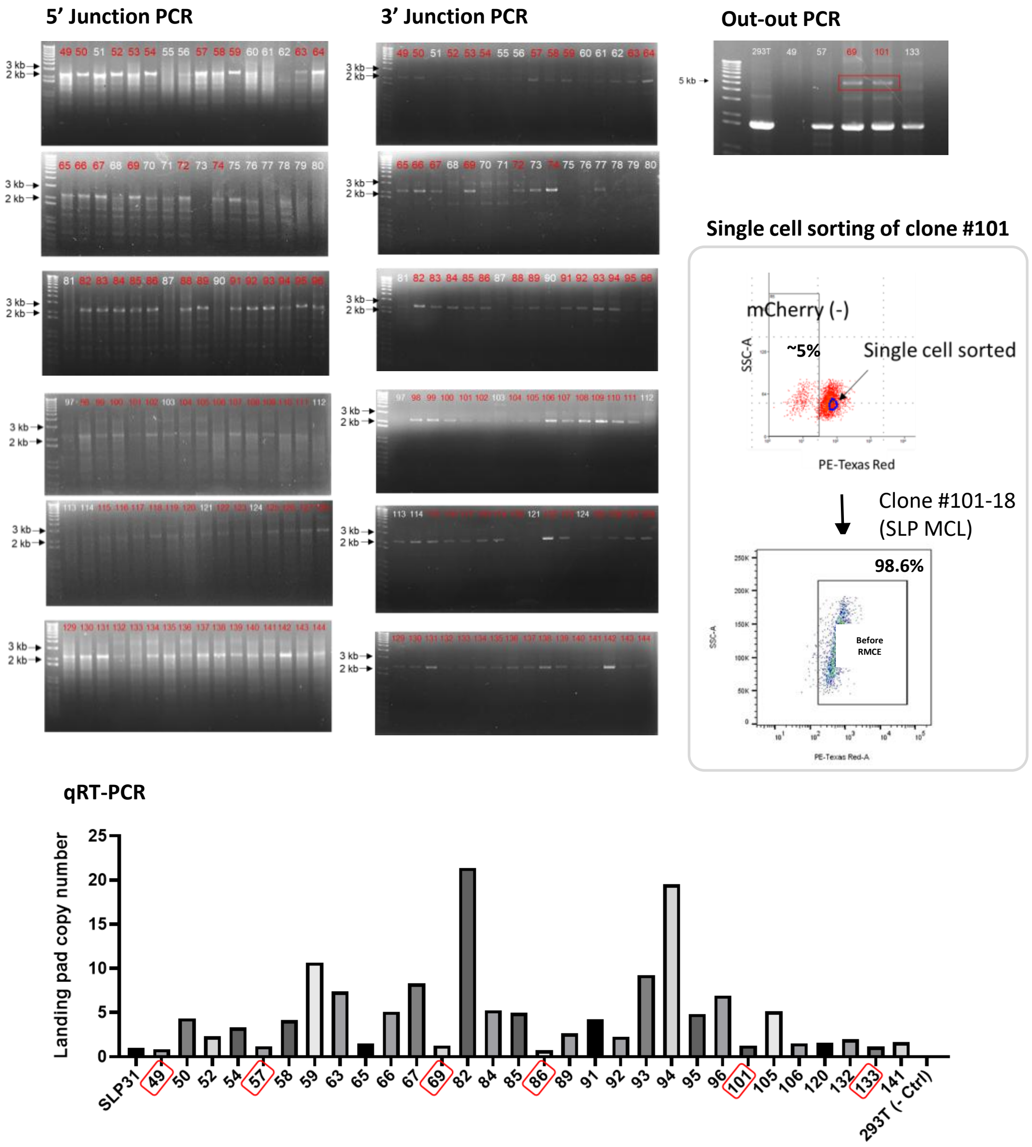

Figure S1. Targeted integration at the *ROSA26* locus was verified in 96 clones through 5'/3'-junction polymerase chain reaction (PCR), with positive clones highlighted in red. Following junction PCR, quantitative real-time PCR (qRT-PCR) was performed to confirm single-copy integration of the landing pad, using the reference single landing pad (SLP)31 clone for comparison. Among SLP clones identified, two clones were positive for out-out PCR. Clone #101 was initially selected based on its superior growth profile and mCherry enrichment; however, approximately 5% of cells were mCherry-negative. To improve population homogeneity, the mCherry-positive fraction was single-cell sorted, and subclones were evaluated. Clone #101-18 (SLP101-18) was selected as the master cell line (MCL) for further applications, as it exhibited a reduced mCherry-negative fraction (~1–2%) while maintaining robust growth.

#101-18 (D11)
NTC

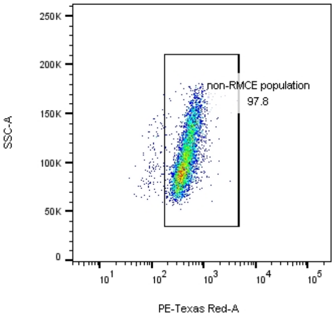

Non-RMCE population: 97.8 %

#101-18 (D11)
RMCE

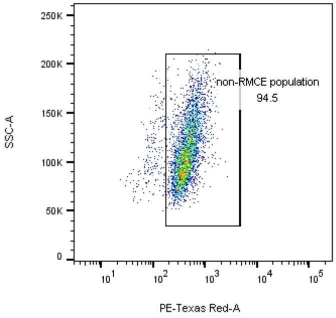

Non-RMCE population: 94.5 %

RMCE efficiency: 3.3 %

Figure S2. RMCE efficiency test of single landing pad master cell line (clone #101-18). Cells were transfected with gRNA library plasmid and NP-C-NLS-Bxb1 plasmid at a 3:1 weight ratio using PEIMAX as previously described (1). 48 hours-post transfection (HPT), cells were passaged every 3 days with fresh media. RMCE efficiency was measured at day 11 after transfection.

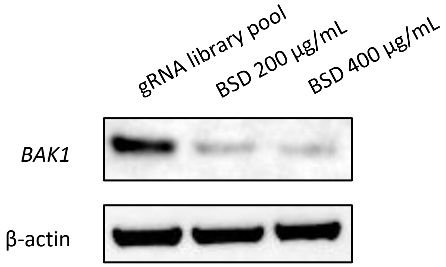

Figure S3. Knockout efficiency test of HEK293T cell-based gRNA library. For transfection, cells were seeded at a concentration of 1 × 10^6 cells/mL in fresh medium and transfected with gRNA(*BAK1*)-Cas9-T2A-BSD vector and PEIMAX (Polysciences, Warrington, PA) as previously described (1). 48 HPT, cells were selected with 200 and 400 µg/mL blasticidin for 3 days. After selection, cells were recovered for 4 passages. β-actin was used as a loading control.

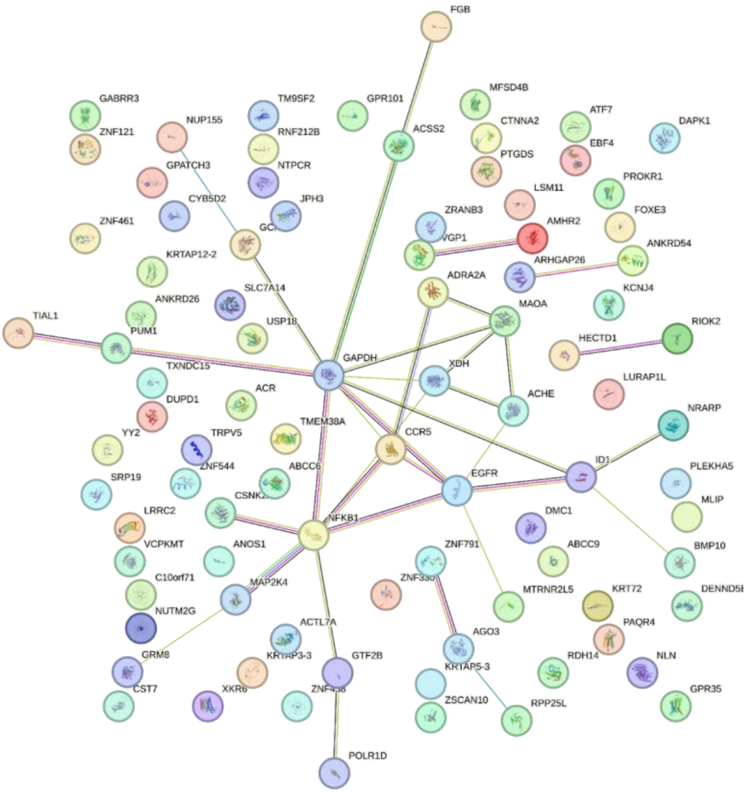

Figure S4. Schematic illustration of protein-protein interaction by STRING analysis of 93 genes (≥2 sgRNA per gene).

HEK293T

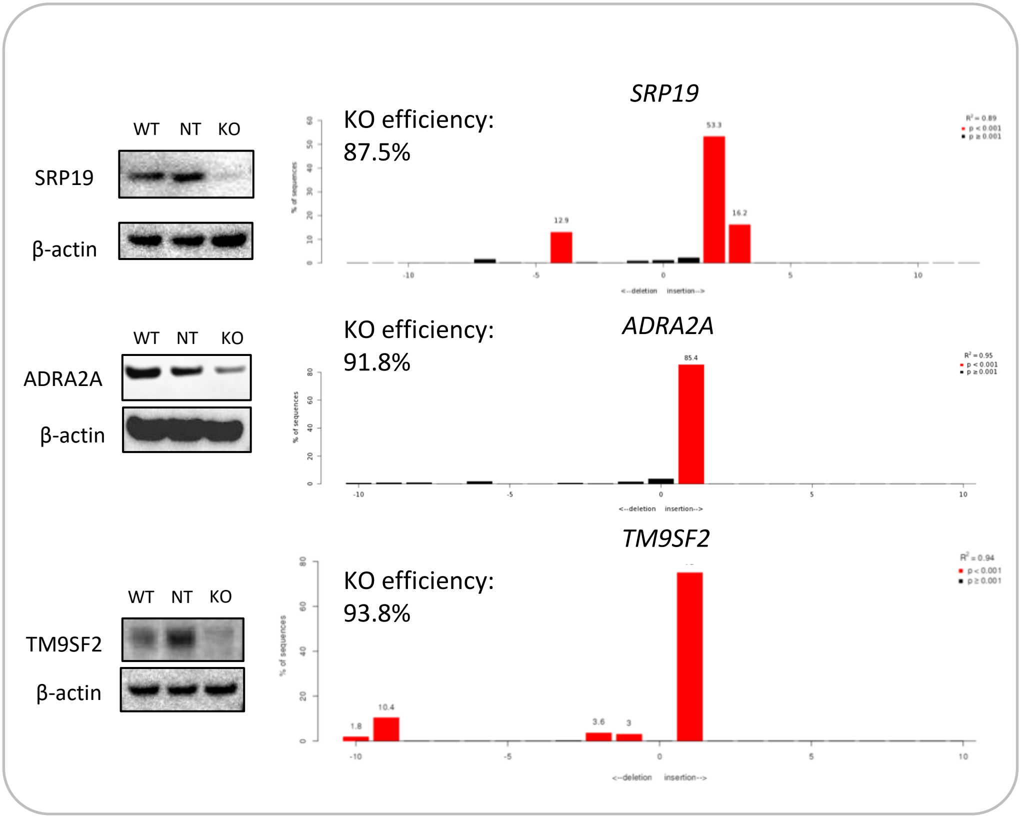

HEK293

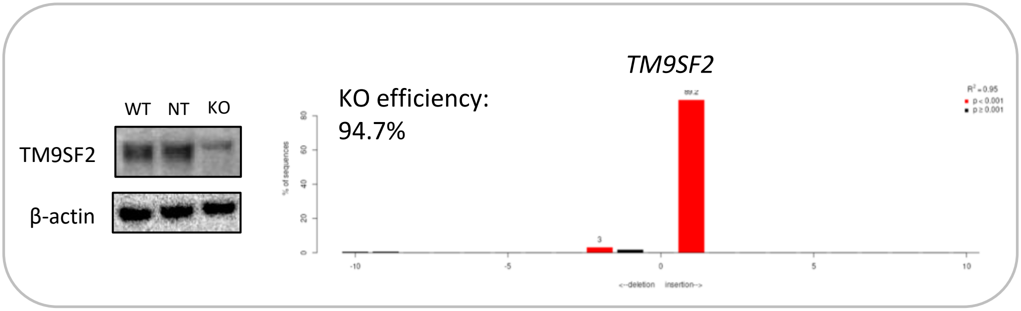

Figure S5. Evaluation of genome editing efficiency in knockout cell pools through western blotting and tracking of indels by decomposition (TIDE). Western blotting and TIDE analysis were conducted as previously described.(40)

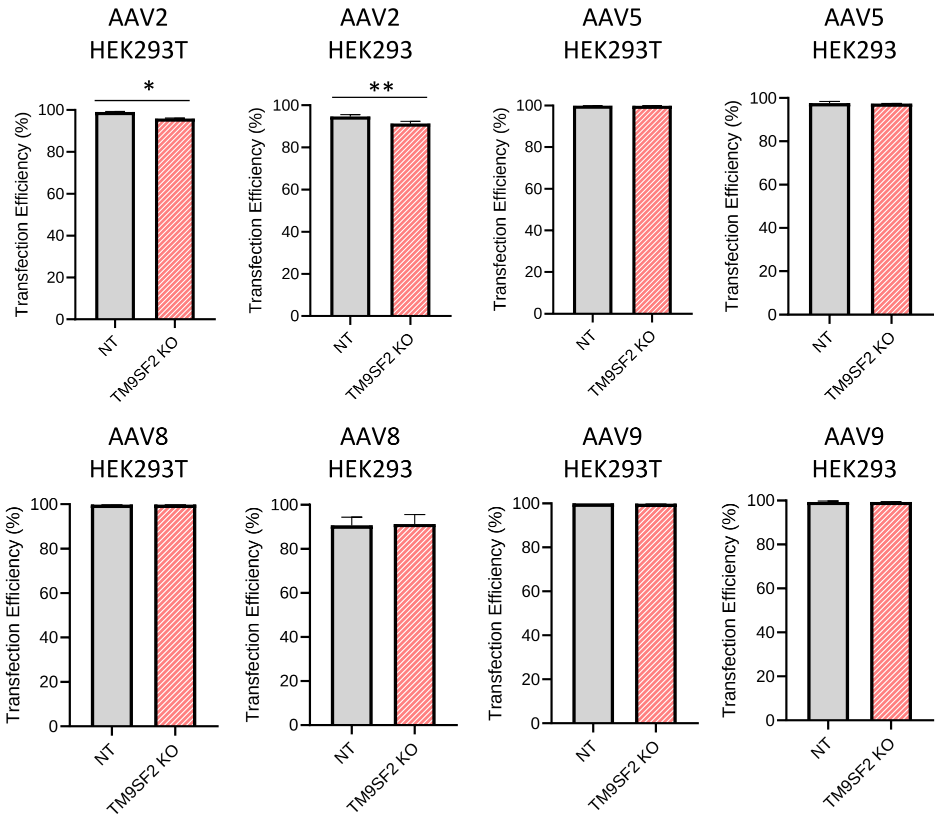

Figure S6. Transfection Efficiency During AAV Production

Table S1. Top 10 candidate genes from 93 significant genes (≥2 sgRNAs per gene).

Gene	Gene name	aRRA	# sgRNAs	# signif. sgRNAs
SRP19	signal recognition particle 19	3.78E-05	4	2
ADRA2A	adrenoceptor alpha 2A	7.55E-05	4	2
EGFR	epidermal growth factor receptor	7.58E-05	4	3
TM9SF2	transmembrane 9 superfamily member 2	0.0001	4	2
ZNF791	zinc finger protein 791	0.000121	4	2
ACHE	acetylcholinesterase	0.000138	4	2
PROKR1	prokineticin receptor 1	0.000163	4	2
TXNDC15	thioredoxin domain containing 15	0.000201	4	2
ACR	acrosin	0.000202	4	2
KRTAP5-3	keratin associated protein 5-3	0.00022	4	2

Table S2. Plasmid information.

Plasmid name	Description	Reference
ROSA26 LP donor	Landing pad donor plasmid targeting *ROSA26* locus	Shin et al., 2020
pU6-(BbsI)_CBh-Cas9-T2A-BFP vector	BFP-all-in-one CRISPR/Cas9 plasmid	Addgene plasmid #64323
pU6-(*ROSA26*)_CBh-Cas9-T2A-BFP	BFP-all-in-one CRISPR/Cas9 plasmid targeting *ROSA26* site	Cloned in this study
PuroR-attB-Esp3I	gRNA library backbone plasmid,<br>Plasmid for RMCE efficiency test	Kim et al., 2023
NP-C-NLS-Bxb1	Bxb1 recombinase vector with nucleoplasmin signal sequence in C terminus	Shin et al., 2021
gRNA(*BAK1*)-Cas9-T2A-Blast	KO efficiency test for deteriminig BSD concentration	Cloned in our previous study (Manuscript in revision)
Cas9-Blast	Cas9 expression plasmid that includes a blasticidin resistance gene.	Xiong et al., 2021<br>Kim et al., 2023
pSpCas9(BB)-T2A-Hygro	Hygro-all-in-one CRISPR/Cas9 plasmid	Addgene plasmid # 127763
gRNA(*target gene*)-Cas9-T2A-Hygro	All-in-one CRISPR/Cas9 plasmid targeting candidate KO target gene	Cloned in this study
pHelper	A vector containing the helper genes (E2A, E4orf6, and VA) for AAV production	A kind gift from Stratagene
pAAV2/2	A vector containing the AAV2 Rep gene and AAV2 Cap gene	Addgene plasmid #104963
pAAV2/5	A vector containing the AAV2 Rep gene and AAV5 Cap gene	Addgene plasmid #104964
pAAV2/8	A vector containing the AAV2 Rep gene and AAV8 Cap gene	Addgene plasmid #112864
pAAV2/9n	A vector containing the AAV2 Rep gene and AAV9 Cap gene	Addgene plasmid #112865

LP, landing pad; RMCE, recombinase-mediated cassette exchange; NP, Nucleoplasmin; NLS, nuclear localization signal; Blast, blasticidin resistance gene; Hygro, hygromycin resistance gene

Table S3. Primer information.

**Junction PCR primers**	
5‘ junction fwd	AAACCGGACGGAGCCAT
5‘ junction rev	CCATGTTATCCTCCTCGCCC
3‘ junction fwd	ATTCCCAATACGAGGTCGCC
3‘ junction rev	AAGGCATGACCAAAGGGGAG
**Outout PCR primers**	
Outout fwd (Rosa26 LP Seq 2)	GCTGCAATCCTGAGGGA
Outout rev (3' junction rev)	AAGGCATGACCAAAGGGGAG
**qRT-PCR primers**	
qRT-PCR fwd (mCherry)	GACTACTTGAAGCTGTCCTTCC
qRT-PCR rev (mCherry)	CGCAGCTTCACCTTGTAGAT
ACTB fwd	CTGGAACGGTGAAGGTGACA
ACTB rev	AAGGGACTTCCTGTAACAACGCA
AAV qRT-PCR fwd	ATGGTGATGCGGTTTTGGCAG
AAV qRT-PCR rev	GGCGGAGTTGTTACGACATTTTGG
**NGS primers**	
NGS fwd (gDNA)	GCTTTATATATCTTGTGGAAAGGACGAAACACC
NGS rev (gDNA)	CCGACTCGGTGCCACTTTTTCAA

Table S4. sgRNA oligo information.

Oligo name	Sequence (5' – 3')
*ROSA26*Fwd	CACCGGTCGAGTCGCTTCTCGATTA
*ROSA26*Rev	AAACTAATCGAGAAGCGACTCGACC
*BAK1*Fwd	CACCGGTTGATGTCGTCCCCGATGA
*BAK1*Rev	AAACTCATCGGGGACGACATCAACC
SRP19Fwd	CACCGTCTTGCTTACCTTACTTATG
SRP19Rev	AAACCATAAGTAAGGTAAGCAAGAC
ADRA2AFwd	CACCGTGGTCGTTGATCTCGCAGCG
ADRA2ARev	AAACCGCTGCGAGATCAACGACCAC
EGFRFwd	CACCGGTCTGCGTACTTCCAGACCA
EGFRRev	AAACTGGTCTGGAAGTACGCAGACC
TM9SF2Fwd	CACCGACACTTACATAGAGTCACAA
TM9SF2Rev	AAACTTGTGACTCTATGTAAGTGTC
ZNF791Fwd	CACCGTCACGTGTACTCGAAAGGCT
ZNF791Rev	AAACAGCCTTTCGAGTACACGTGAC
ACHEFwd	CACCGTGTCCTCGTCTGGATCTATG
ACHERev	AAACCATAGATCCAGACGAGGACAC
PROKR1Fwd	CACCGAGTGCGCAGGTAGTTGACAG
PROKR1Rev	AAACCTGTCAACTACCTGCGCACTC
TXNDC15Fwd	CACCGGGACACCCAAGGCGATCACA
TXNDC15Rev	AAACTGTGATCGCCTTGGGTGTCCC
ACRFwd	CACCGAAGCGCCCACACGAAATGGG
ACRRev	AAACCCCATTTCGTGTGGGCGCTTC
KRTAP5-3Fwd	CACCGGCAGACAGGTACACAGCAGC
KRTAP5-3Rev	AAACGCTGCTGTGTACCTGTCTGCC

Table S5. Significant genes.

Table S6. GO stat.

Table S7. GO genes.

Table S8. KEGG stat.

Table S9. KEGG genes.
